# Coumarins Disrupt Cell–Cell Communication and Virulence in Priority Pathogens: Targeting the PQS Signalling System in *Pseudomonas aeruginosa*

**DOI:** 10.1111/1751-7915.70404

**Published:** 2026-06-26

**Authors:** Dylan Boon, Benjamin O'Rourke, Muireann Carmody, David F. Woods, Antje Gloe, Daniel Platero‐Rochart, Pedro A. Sánchez‐Murcia, Gerard P. McGlacken, F. Jerry Reen

**Affiliations:** ^1^ School of Microbiology University College Cork Cork Ireland; ^2^ School of Chemistry and Analytical & Biological Chemistry Research Facility (ABCRF), University College Cork Ireland; ^3^ Institute for Pharmaceutical Microbiology University of Bonn Bonn Germany; ^4^ Laboratory of Computer‐Aided Molecular Design, Division of Medicinal Chemistry, Otto‐Loewi Research Center Medical University of Graz Graz Austria; ^5^ BioTechMed‐Graz Graz Austria; ^6^ SSPC The Research Ireland Centre for Pharmaceuticals Ireland

## Abstract

Cell‐to‐cell communication in microbial systems is known for its vital role in cellular signalling and gene expression. A specific form termed Quorum Sensing (QS) has received considerable attention since its discovery in the marine symbiont *Aliivibrio fischeri* . QS‐controlled microbial functions are associated with bacterial virulence, pathogenicity, host–microbe interactions, and biofilm development. Interference in these signalling systems can modulate microbial virulence and pathogenicity, and microbial infection caused by drug‐resistant pathogens. Plant‐derived phytochemicals are considered a promising candidate, with coumarins emerging as significant plant‐derived signalling molecules shaping microbiome dynamics and pathogen behaviours from a broad spectrum of ecosystems. Here we explored the role of natural and synthetic coumarin compounds in the control of signalling and virulence traits in *Pseudomonas aeruginosa* and other priority bacterial pathogens, including the fungal opportunist *Aspergillus fumigatus* . We uncovered an important ‘hydroxylation‐bias’ favouring coumarin, umbelliferone (7‐OH), and 6‐hydroxy‐coumarin (6‐OH) in the specific competitive inhibition of the Pseudomonas Quinolone Signal (PQS), associated with reduced activity of a PqsR translational fusion and suppression of pyocyanin production. Conversely, while esculetin (6,7‐OH) was most effective at Acyl Homoserine Lactone (AHL) QS biosensor inhibition, it did not affect PQS production. Anti‐biofilm activity of coumarins against *P. aeruginosa* was independent of initial attachment but linked to changes in exopolysaccharide production. As the very real threat posed by antimicrobial resistance persists, these data support a role for phytochemicals such as coumarins in delivering an ecological solution to dysbiosis in the host–microbe interaction.

## Graphical abstract

The selective nature through which coumarin compounds produced by different species of plants target PQS signalling in *Pseudomonas aeruginosa and* select for distinct phenotypic responses in key pathogenic organisms adds to the growing evidence of their utility as natural virulence suppressors.

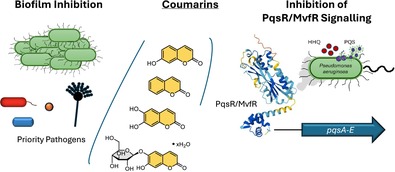

## 1 Introduction

Important polyphenolic plant metabolites classed as coumarins have recently emerged as key phytochemicals with the potential to shape microbial communities in the rhizosphere (Niro et al. 2016; Tsai and Schmidt 2017; Stringlis et al. 2019; Voges et al. 2019; Stassen et al. 2021). The manner in which different cultivars of plant host select for specific microbes in the rhizosphere reflects an exquisite level of intricacy in the host–microbe interaction and in the requirements each distinct cultivar may have for its optimal growth and propagation (Mark et al. 2005; Fitzpatrick et al. 2018). Recently, a mechanistic insight into the coumarin‐plant‐microbiome relationship has come in the form of MYB72, a host transcription factor, and BGLU42, a glucosidase involved in processing the glycoside form of coumarins prior to release from the plant (Stringlis et al. 2018). It has also been proposed that mobilisation of iron by plant‐derived coumarins may underpin, at least in part, the tailoring of the rhizosphere microbiome (Niro et al. 2016; Tsai and Schmidt 2017; Stringlis et al. 2018, 2019; Voges et al. 2019; Stassen et al. 2021). However, the extent to which plant‐species interactions are governed by coumarin‐mediated control of colonisation and polymicrobial interactions remains to be established.

Separately, the growing concern over the inexorable spread of antimicrobial resistance and the parallel drop off in the development of novel antimicrobial compounds has underlined the need for alternative strategies for control of microbial behaviour (Cooper and Shlaes 2011; Prestinaci et al. 2015). In recent years, there has been a shift towards research into molecules that can intercept the virulence and pathogenic behaviour of microbes, without targeting growth per se. The concept of anti‐virulence has been tested in a range of bacterial and fungal pathogens with some degree of success achieved in vitro and in vivo in the case of small molecules (Mlot 2009; Rampioni et al. 2014; Dickey et al. 2017; Whiteley et al. 2017; Grossman et al. 2020; Jonkergouw et al. 2023; Lu et al. 2025; Zhou et al. 2025), and more recently monoclonal antibodies (Yang et al. 2015; Wilcox et al. 2017; Jurcisek et al. 2022). The prominent role of coumarins in moderating microbiome dynamics in the rhizosphere suggests that similar structures may be effective in modulating human pathogenic microbes. It remains unclear in this regard whether they act as coercive compounds or rather as cues or signals targeting specific communication systems directly. Elegantly described by Diggle and colleagues, the distinction is an important one (Diggle et al. 2007).

Several clinically approved drugs contain the coumarin moiety. Amongst the most notable are anticoagulants such as warfarin, acenocoumarol and phenprocoumon, which act as vitamin K antagonists and are widely used for the prevention and treatment of thromboembolic disorders. Coumarin derivatives are also found in tioclomarol and dicoumarol, which exhibit similar anticoagulant properties. While not widely used as a drug due to potential hepatotoxicity, coumarin itself has been studied for various pharmacological effects, including anti‐inflammatory and anticancer properties. The inclusion of the coumarin core in these agents highlights its importance as a versatile pharmacophore in medicinal chemistry. Coumarin compounds have also been explored as potential combinatorial adjuncts for antibiotic treatments, with some evidence suggesting synergistic effects observed against a range of multidrug resistant pathogens (Zuo et al. 2016; Madeiro et al. 2017; Feng et al. 2020). The mechanism of action underlying antibacterial activity or antibiotic potentiation has not yet been uncovered, though possible roles for quorum sensing, dihydrofolate reductase, and efflux have all been reported (Gutiérrez‐Barranquero et al. 2015; Zhang et al. 2018; Bhagat et al. 2019; Feng et al. 2020; Qais et al. 2021; Tajani et al. 2023).

The natural role of coumarins in moderating the behaviour of microbial species led us to explore the chemical diversity of plant‐derived coumarins exerting control over key virulence and pathogenesis phenotypes in clinically relevant pathogens, both bacterial and fungal. In particular, we focused on species of bacteria that pose the greatest risk in the emergence of antimicrobial resistance (AMR), most of which are members of the ESKAPEE pathogen group (Ngoi et al. 2021). Recognising the challenges posed by fungal colonisation in immunocompromised patients, *Aspergillus fumigatus* was also included in the analysis (Casalini et al. 2024). Previous work has suggested that distinct coumarin molecules can exert control over biofilm formation, cell–cell communication, secretion and growth (Brackman et al. 2009; Lee et al. 2014; Gutiérrez‐Barranquero et al. 2015; D'Almeida et al. 2017; Pan et al. 2017; Reen, Gutiérrez‐Barranquero, et al. 2018; Zhang et al. 2018; Qin et al. 2020; Qu et al. 2020; Thakur et al. 2020; Qais et al. 2021; Alqarni et al. 2022; Chadha et al. 2022; He et al. 2022; Abdelaziz et al. 2023; Martin et al. 2023; Tajani et al. 2023; Ahmed et al. 2024; Pakeeraiah et al. 2024; Lu et al. 2025), though a universal understanding of the mechanism underpinning these effects in bacterial and fungal pathogens remains elusive. Therefore, we explored a broader role for natural coumarins in moderating pathogen control, uncovering key structural and mechanistic insights that support a role for coumarins as a plant‐derived intervention against fungal opportunists and a subset of bacterial ESKAPEEs. Exquisite structural specificity was identified in the control of Pseudomonas Quinolone Signal (PQS) production, an important interspecies/interkingdom communication molecule and core element of quorum sensing (QS) in *P. aeruginosa* . Molecular Dynamics (MD) simulations supported a role for coumarin and umbelliferone as inhibitors of signal binding to the PqsR (but not LasR) QS regulators, with further analysis revealing this inhibition to be competitive. Taken together, these data provide a new mechanistic insight into the action of the coumarin class of phytocompound towards pathogenic microbes, and it will form the basis of future studies to explore the functionality of these molecules in polymicrobial communities.

## 2 Experimental Procedures

### 2.1 Bacteria and Culture Conditions

*Pseudomonas aeruginosa* and *Escherichia coli* were grown in Lysogeny Broth (LB) media while *Staphylococcus aureus* and *Staphylococcus haemolyticus* were grown in Tryptic Soy media (TSA or TSB, Merck). All bacteria were incubated while shaking at 150 rpm at 37°C. Coumarin compounds (added at T_0_ for all experiments) used in this study were dissolved in Dimethyl Sulfoxide (DMSO, Sigma‐Aldrich) and stored at 4°C. *Aspergillus fumigatus* was routinely grown on *Aspergillus* Minimal Medium (AMM) agar and spores were collected in PBS supplemented with 0.05% (v/v) Tween 20.

### 2.2 Quorum‐Sensing Inhibition Assays

*Serratia* sp. SP15 (a derivative of the ATCC 39006 strain, inhibition of short‐chain AHLs, e.g., C4‐HSL) and *Chromobacterium violaceum* DSM30191 (inhibition of short‐medium chain AHLs, e.g., C4‐C8‐HSL) (Slater et al. 2003; Morohoshi et al. 2008; Poulter et al. 2010) were spread over LB agar plates at an OD_600nm_ of 0.5 using cotton swabs which had been dipped in the respective cultures. Wells were punctured into the agar plates and the coumarin compounds (50 μL aliquots) were added at total mass of 200, 150, 100 or 50 μg. All biosensor strains were incubated at 30°C for 24 h and the effect on colour and growth was documented. Phosphate buffered saline (PBS) acted as the control. To assess metabolic activity in the zones of inhibition, 20 μL of XTT [2,3‐bis‐(2‐methoxy‐4‐nitro‐5‐sulfophenyl)‐2H‐tetrazolium‐5‐carboxanilide, 0.5 mg ml^−1^; Sigma‐Aldrich] and 1 μM menadione (Sigma‐Aldrich) were added to the zones of inhibition on the QS biosensor agar plates and incubated for 20 min at room temperature as described previously (Woods et al. 2022). Metabolic activity was qualitatively assessed through colourimetric inspection. The presence of a red pigment surrounding the test wells was considered positive for active cells.

### 2.3 Biofilm Assays

#### 2.3.1 Microtitre Based Biofilm Assays

Overnight bacterial cultures were prepared in fresh media at OD_600nm_ 0.05 and were transferred in quadruplicate 200 μL aliquots into flat‐bottomed 96‐well plates (Cellstar, Sigma Aldrich) with the respective coumarin treatments. *A. fumigatus* spores were inoculated at 10^5^ spores/ml into AMM media and similarly transferred. In each case, the microtitre plates were incubated statically at 37°C for 20 h. The liquid cultures were removed following incubation, plates were rinsed with sterile deionised water and allowed to air dry for 20 min. Wells were stained with 200 μL of 0.1% (w/v) crystal violet (Sigma‐Aldrich) for 30 min and then washed in deionised water to remove unbound crystal violet. The bound stain was eluted by addition of 200 μL of 96% ethanol. Biofilm formation was monitored and quantified after 20 min by measuring the absorbance at Abs_595nm_ with a Thermo Scientific Multiskan GO Microplate Spectrophotometer. Time course biofilm assays were performed as above with each time point being prepared as a single plate. Plates were processed as above at 2, 4, 6, 8 and 10 h. For microscopic analysis of fungal biofilms, *A. fumigatus* spores were inoculated at 10^5^ spores/ml into AMM media and transferred in triplicate 1 mL aliquots into 24‐well plates with a sterile coverslip. After static incubation for 20 h, the coverslips were removed, washed with PBS, and stained with crystal violet for 30 min. Further washing with distilled water removed background stain and enabled visualisation under a light microscope at 400X magnification.

#### 2.3.2 Minimum Inhibitory Concentration (MIC) Biofilm Assays

MIC biofilm assays were carried out in standard 96 well plates. *P. aeruginosa* PA14 was inoculated overnight in LB at 37°C shaking. The wells were first filled with 100 μL LB media and compounds were added at a concentration of 32 mM in 100 μL. Serial dilution was carried creating a range from 8 mM to 15.625 μM. The cultures were added in 100 μL volumes at a starting OD_600nm_ of 0.05, to a total volume of 200 μL in each well. Plates were incubated overnight for 20 h at 37°C, after which all liquid was removed from the wells, and they were rinsed with water. Crystal violet staining and quantification was performed as described above.

#### 2.3.3 Attachment Assays

Attachment assays were performed as above with the exception that the starting OD_600nm_ of 0.25 was used and plates were incubated for 2 h prior to processing.

#### 2.3.4 EPS Production Assays

Exopolysaccharide (EPS) production was measured on LB media in 6‐well plates supplemented with 20 μg/mL of Coomassie Brilliant Blue and 40 μg/mL of Congo red (Gupta and Schuster 2012; Shatila et al. 2021). Each coumarin compound was added to a final concentration of 2 mM and an inoculum of the overnight culture was spotted on the centre of each well. Plates were incubated for 120 h at 37°C and visualised daily as the staining of cells developed. In addition to testing PAO1 and PA14 strains, an additional mutant strain of PA14 sourced from the non‐redundant library was included (*tbpA*, *PA14_13660*), based on its constitutive EPS positive phenotype.

### 2.4 Growth Curve Analysis

Bacteria were inoculated at 0.05 OD_600nm_ in respective liquid media with coumarin compounds at a final concentration of 2 mM. The bacterial growth was monitored in 100‐well honeycomb plates on a BioScreen C instrument (Labsystems Oy Ltd). Compound **8** (7‐amino‐4‐trifluromethyl‐2‐coumarin) is a dye, and interference with the automated OD_600nm_ readings led us to adopt a viable cell count protocol. Glass universals containing 5 mL LB with a starting of 0.05 and a concentration of 2 mM compound 10 were incubated at 37°C with 150 rpm shaking. At 4‐, 8‐ and 12‐h time points, dilutions in PBS were plated on LB with DMSO acting as a control.

### 2.5 Promoter Fusion Analysis

Cells from overnight *P. aeruginosa* cultures carrying the appropriate promoter fusion (Table S1) were transferred into fresh media at OD_600nm_ 0.02 and incubated with shaking at 37°C. Cells were sampled at intervals to assess promoter activity using the Miller Assay described previously (Reen, Phelan, Woods, et al. 2016). Miller assays performed with compound **8** were conducted using viable cell counts and are presented as Miller Equivalents where log10 (cfu/mL) replaces OD_600nm_ in the calculation.

### 2.6 Pyocyanin Production

Conical flasks containing 25 mL of LB broth were inoculated with *P. aeruginosa* at an OD_600nm_ of 0.05 and supplemented with 2 mM of each compound. LB broth with DMSO and untreated culture were used as controls. Following incubation of 20 h at 37°C with shaking at 180 rpm, 5 mL of culture was removed and centrifuged at 5000 *g* for 7 min. Pyocyanin was then extracted as described previously (Essar et al. 1990). The concentration (μg/mL) was calculated using the following formula: concentration (μg/mL) = Abs_520nm_ × 17.072 × dilution factor.

### 2.7 Pseudomonas Quinolone Signal (PQS) and 2‐Heptyl‐4‐Quinolone (HHQ) Extraction and TLC Analysis

Overnight cultures of *P. aeruginosa* PAO1 were transferred at a starting OD_600nm_ of 0.05 to 25 mL of LB media in conical flasks. Compounds 1 (coumarin), 2 (umbelliferone) and 3 (esculetin) were introduced at a final concentration of 2 mM, DMSO and untreated culture acted as controls. The conical flasks were incubated at 37°C at 180 rpm. After 8 h, 10 mL of the culture was removed from which an organic extraction of HHQ and PQS was performed and analysed by Thin Layer Chromatography (TLC) using Silica gel 60 F_254_ plates (Merck) according to the protocols established by Fletcher and colleagues (Fletcher et al. 2007). The OD_600nm_ was comparable across all treated samples compared to the DMSO control ensuring standardisation of extraction outputs.

### 2.8 Molecular Modelling of Coumarins at the PqsR and LasR Receptors

The Cartesian coordinates of the two transcriptional regulators from *P. aeruginosa* PqsR (UNIPROT id. Q9I4X0) and LasR (UNIPROT id. P25084) were obtained from the crystallographic structures of PqsR in complex with triazolo‐pyridine inverse agonist A (PDB id. 6YIZ) (Schütz et al. 2021) and LasR in complex with 3‐oxo‐C12‐HSL (PDB id. 2UV0) (Bottomley et al. 2007), respectively. A detailed description of the methodology applied to docking and modelling of the coumarins and LasR or PqsR interaction is described in Methods File S1. Docking studies were performed with a grid of 60 × 60 × 60 Å dimensions (Klett et al. 2012), using the genetic algorithm (GA) implemented in AutoDock4 (Morris et al. 2009). These structures were used for further MD simulation studies. The protein residues were described with the AMBER force field parameters ff19SB (Tian et al. 2020) and the atoms of the small molecules were described as GAFF/AMBER atom types (Vosko et al. 1980; Lee et al. 1988; Becke 1993; Devlin et al. 1995). All proteins were embedded in a truncated octahedron of water molecules (TIP3P) (Jorgensen et al. 1983) and two different setups were built: (a) the docking solution as a protein monomer with a small single molecule at the binding site; (b) the protein with 10 (PqsR, protein monomer) or 20 (LasR, as protein dimer) small molecules in the solution but not in the binding site from the beginning. Long‐range interactions were calculated using Particle Mesh Ewald summations (Essmann et al. 1995) using Periodic Boundary Conditions and a cutoff of 10 Å for non‐bonded interactions. All MD simulations were run using the suite of programs Amber20 (Case et al. 2023) and the MD trajectories were analysed using cpptraj v5.1.0 (Roe and Cheatham 2013). Pocket and tunnel screening was performed using the Caver web server, predicting the first possible pockets in the apo form of both proteins (Stourac et al. 2019). Caver default parameters were used in all cases.

### 2.9 Chemical Synthesis

A detailed description of the chemical synthesis performed in this study, including 1H NMR, 19F NMR and 13C NMR spectra, is provided in Methods File S2.

### 2.10 Statistical Analysis

All experiments performed contained a minimum of three independent biological replicates. Statistical analysis was carried out on the GraphPad software using one‐way ANOVA testing followed by Dunnett's multiple comparison. Differences were considered significant when the *p*‐value was ≤ 0.05 as indicated by an asterisk.

## 3 Results

### 3.1 Natural Coumarins Exhibit Specific Patterns of Quorum Sensing (QS) Inhibition in AHL Biosensors

**FIGURE 1:**
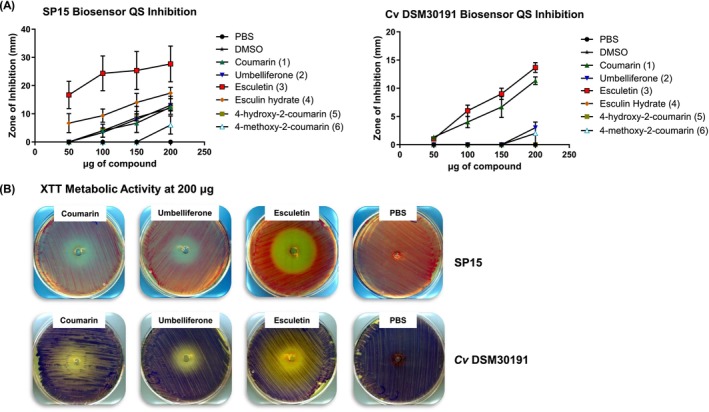
Quorum sensing inhibition by coumarin compounds in AHL‐biosensor strains ((1) Coumarin; (2) Umbelliferone; (3) Esculetin; (4) Esculin hydrate; (5) 4‐hydroxy‐2‐coumarin; (6) 4‐methoxy‐2‐coumarin, as per Table 1). (A) Zone of inhibition from *S. marcescens* and *C. violaceum* biosensors. Data presented is the mean (±SEM) of three independent biological replicates. (B) Visualisation of XTT metabolic activity in the zone of clearing for the biosensors at 200 μg/mL or coumarin compound. Data presented is representative of three independent biological replicates.

Testing the Quorum Sensing Inhibition (QSI) activity of coumarins towards short chain AHL signalling using *Serratia* sp. SP15, esculetin (**3**) and esculin hydrate (**4**) exhibited QSI activity at 200, 150, 100 and 50 μg, with the former showing the largest zone of pigment reduction (Table 1, Figure 1A and Figure S1). Coumarin (**1**), umbelliferone (**2**), and 4‐hydroxy‐2‐coumarin (**5**) also exhibited zones of inhibition at doses of 100 μg and above. In relation to medium‐chain AHLs tested on *C. violaceum* DSM30191, coumarin (**1**) and esculetin (**3**) inhibited pigmentation at 200, 150 and 100 μg. In contrast, esculin hydrate (**4**) showed no inhibition of pigmentation. Inclusion of XTT in the assays provided an assessment of metabolic activity in the zones of clearance, indicating that the reduction in pigmentation was not simply a result of growth inhibition or metabolic suppression (Figure 1B; Figure S1). XTT activity was observed at all concentrations of esculetin (**3**), though the 200 μg/mL concentration of coumarin (**1**) and umbelliferone (**2**) did lead to a reduction or loss of visible XTT pigmentation.

**TABLE 1:** Coumarin structures, sources and natural sources.

No.	Compound	Chemical name	Structure	Source^a^
**1**	Coumarin	coumarin	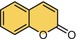	Plants, marine sponges and some microbes
**2**	Umbelliferone	7‐hydroxy‐2‐coumarin	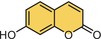	Rutaceae and Apiaceae (e.g., celery)
**3**	Esculetin	6,7‐dihydroxy‐2‐coumarin	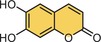	Medicinal plants e.g., *Cichorii flos*
**4**	Esculin hydrate	6‐(β‐bergam‐D‐Glucopyranosyloxy)‐7‐hydroxy‐2‐coumarin	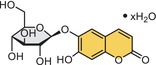	*Aesculus hippocastanum* (horse chestnut)
**5**		4‐hydroxy‐2‐coumarin	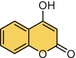	*Ruta graveolens* , *Vitis vinifera* and *Apis cerana*
**6**		4‐methoxy‐2‐coumarin	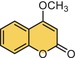	*Ferula assa‐foetida*

No	Analogue	Chemical name	Structure	Source
**7**	Warfarin	4‐Hydroxy‐3‐(3‐oxo‐1‐phenylbutyl)coumarin	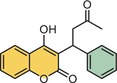	Chemically derived from coumarin.
**8**		7‐amino‐4‐(trifluoromethyl)‐2‐coumarin	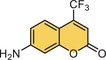	Chemically derived from coumarin.
**9**		6‐methyl‐4‐methoxy‐2‐pyrone	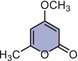	Synthetic
**10**		4‐hydroxy‐6‐methyl‐pyrone	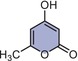	Synthetic

^a^ All compounds sourced from Sigma Aldrich for this study.

### 3.2 Coumarins Suppress Biofilm Formation in *P. aeruginosa* and Other Key Nosocomial Pathogens

**FIGURE 2:**
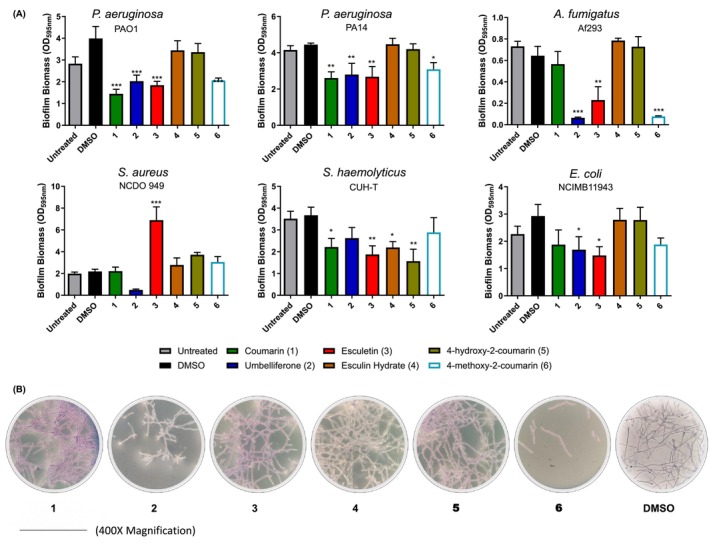
Biofilm formation of microbial pathogens in the presence of coumarin compounds (numbered as per Table 1). (A) Quantitative analysis of coumarins tested at 2 mM concentration. Data presented is the mean (±SEM) of at least three independent biological replicates. Statistical analysis was performed by one‐way ANOVA with Dunnett's post hoc corrective testing (*p ≤ 0.05, **p ≤ 0.005, ***p ≤ 0.001). (B) Microscopic visualisation (400X magnification) of hyphal development in *A. fumigatus* Af293 in the presence of coumarin compounds. Data presented is representative of at least 5 micrographs taken across three independent biological replicates.

Biofilm formation is a major QS‐regulated virulence phenotype in *P. aeruginosa* and other pathogens and previous studies have reported anti‐biofilm properties of several coumarin related structures (Reen, Gutiérrez‐Barranquero, et al. 2018; Sauer et al. 2022). Therefore, the ability of the natural coumarins to interfere with biofilm formation in a range of pathogens was investigated. For *P. aeruginosa* PAO1 there was significant antibiofilm activity (*p* ≤ 0.05) compared to the DMSO control in the presence of coumarin, umbelliferone, esculetin and 4‐methoxy‐2‐coumarin (**6**) (Figure 2). No significant difference or potential inhibitory effect was seen in the presence of esculin hydrate or 4‐hydroxy‐2‐coumarin. In *P. aeruginosa* PA14, reduction in biofilm formation was seen in the presence of the same compounds, with the exception of 4‐methoxy‐2‐coumarin.

Extending the investigation to other pathogens, the species‐specific nature of coumarin activity was evident in the contrasting activity against biofilm formation in *Staphylococcus* species. Esculetin significantly (*p* < 0.0001) enhanced biofilm formation in the NCDO949 strain of *S. aureus* , while it inhibited biofilm formation in a clinical *S. haemolyticus* CUH‐T strain (Figure 2A). In the case of *E. coli* , both umbelliferone and esculetin were found to inhibit biofilm formation (Figure 2A). Umbelliferone, esculetin and 4‐methoxy‐2‐coumarin were able to suppress biofilm formation in the fungal pathogen *A. fumigatus* Af293, whereas coumarin was ineffective (Figure 2A,B). Microscopic imaging revealed that hyphal structures were largely unaffected in the presence of coumarin compounds, with the notable exception of those that reduced biofilm biomass (Figure 2B).

A dose dependent effect was evident in several of the test strains, where at 1 mM coumarin, umbelliferone and esculetin continued to have inhibitory properties on biofilm against *P. aeruginosa* PAO1, while only umbelliferone and esculetin retained activity against the PA14 strain (Figure S2). No inhibitory effect was evident against any of the tested strains at concentrations of 0.1 or 0.01 μM (Figure S2). MIC assays determined an IC_50_ of 3 mM for coumarin, 3.2 mM for umbelliferone, and 3.7 mM for esculetin against *P. aeruginosa* PA14 (Figure S3).

### 3.3 Modulation of Exopolysaccharide (EPS) Production by Specific Natural Coumarins

**FIGURE 3:**
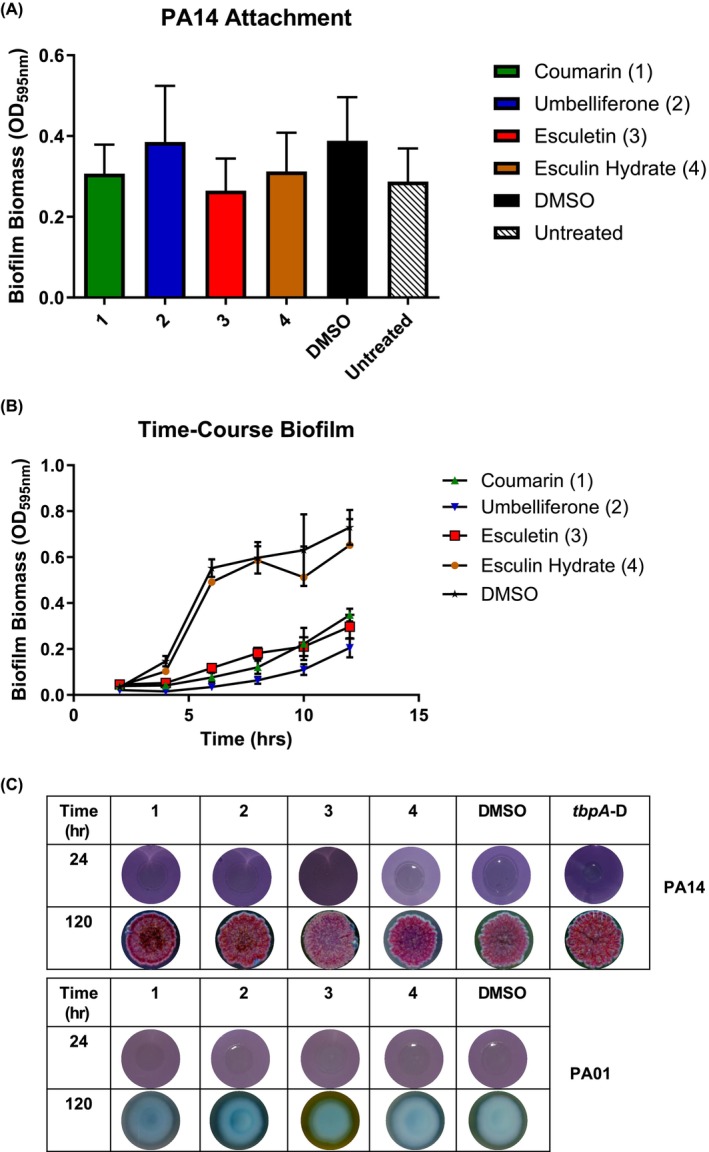
Biofilm (attachment and time‐scale) analysis of *P. aeruginosa* PA14 in the presence of selected coumarin compounds (numbered as per Table 1). (A) Attachment analysis performed over 2 h on microtitre plates. (B) Time‐course biofilm kinetics measuring biomass over a 10 h period from the point of inoculation. (C) Qualitative EPS production measured over 120 h in the presence of selected coumarin compounds. All data presented are the mean (±SEM) of at least three independent biological replicates. Statistical analysis was performed by one‐way ANOVA with Dunnett's post hoc corrective testing.

Biofilm formation is a multi‐stage process and interference with it can occur at distinct phases. The *P. aeruginosa* PA14 strain was selected for analysis based on its strong biofilm‐forming properties and its well characterised EPS morphology. Initial attachment in the reversible phase was unaffected indicating that inhibition of biofilm biomass occurs downstream of microcolony formation (Figure 3A). Assessing biofilm formation over time, it became apparent that the suppression phenotype occurs between 2 and 4 h post inoculation, with a significant increase in biofilm biomass occurring in the carrier control, where biofilm biomass remained unchanged in the coumarin treated samples (Figure 3B). We next studied production of exopolysaccharide to determine if EPS was the target of coumarins, thus preventing the mature biofilm from forming (Figure 3C and Figure S4). PA14 EPS production appeared comparable to the DMSO control in the presence of esculetin. While pigmentation and morphology appeared different in the samples treated with coumarin and umbelliferone, with a more intense red colour than observed in the DMSO control, comparable to the *tbpA‐D* mutant, quantification of EPS production will be required to establish whether coumarin compounds specifically target EPS production. While PA14 relies on pel polysaccharide, the PAO1 strain encodes an intact *psl* operon (Colvin et al. 2012). However, in contrast to the PA14 strain, PAO1 did not exhibit any visible evidence of EPS production either in the presence or absence of coumarin compounds at 37°C (Figure 3C).

Growth was also investigated, as a growth limiting activity could also explain the biofilm results. *P. aeruginosa* PAO1 and *S. haemolyticus* CUH‐T had no significant differences in growth when treated with the coumarin compounds (Figure S5). *P. aeruginosa* PA14 entered stationary phase at a lower biomass in the presence of coumarin, umbelliferone and esculetin, while in *S. aureus* NCDO949 coumarin led to a slower growth rate but same final biomass comparative to the DMSO control. For *E. coli* NCIMB11943, there were significant growth differences for the bacteria inoculated in the presence of coumarin, umbelliferone and esculetin. While these growth effects may explain the reduction in biofilm formation for *E. coli*, they indicate that the suppression of biofilm formation in the other pathogens by coumarins is generally a growth independent phenotype.

### 3.4 The *P. aeruginosa* PQS System Is Selectively Suppressed by Specific Coumarin Compounds

**FIGURE 4:**
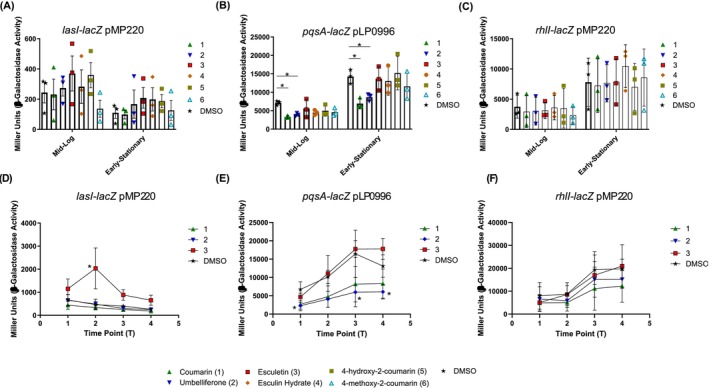
Quorum sensing promoter fusion analysis in *P. aeruginosa* . The three QS systems encoded in *P. aeruginosa* were studied in the presence of coumarin compounds. (A–C) Assays performed in glass vials and (D–F) time‐course assays performed in conical flasks. All data presented is the mean (±SEM) of at least three independent biological replicates. Statistical analysis was performed by one‐way ANOVA with Dunnett's post hoc corrective testing (*p ≤ 0.05).

Previously, coumarin has been shown to suppress the LasIR, RhlIR and PQS QS signalling systems (Gutiérrez‐Barranquero et al. 2015; Reen, Gutiérrez‐Barranquero, et al. 2018; Qais et al. 2021; Tajani et al. 2023). However, the mechanism of action and the role of other coumarin compounds in this regard remain uncharacterised. Promoter activity in PAO1 was initially studied at mid‐log and early stationary phase timepoints (Figure 4A). Interestingly, esculetin led to increased activity at the *lasI* promoter, counterintuitive to what might be expected from the biosensor assays. Both coumarin (mid‐log, *p* = 0.0148) and umbelliferone (mid‐log, *p* = 0.0493) led to a significant suppression of *pqsA* promoter activity in the wild‐type strain, while esculetin and the other coumarins did not influence activity relative to the DMSO control (Figure 4B). Addition of coumarins did not affect promoter activity of *rhlI*, again surprising given that it suppressed QS signalling in the *Serratia* sp. SP15 biosensor (Figure 4C). The activity of coumarin, umbelliferone and esculetin was subsequently assayed over time‐course in shaking flasks to conduct a more detailed kinetic analysis of promoter activity from the QS systems. This confirmed the enhanced *lasI* promoter activity (*p* = 0.0377) in the presence of esculetin (Figure 4D), and the suppression of *pqsA* promoter activity by coumarin and umbelliferone (Figure 4E). No significant change in *rhlI* promoter activity was observed at any of the time points tested (Figure 4F).

**FIGURE 5:**
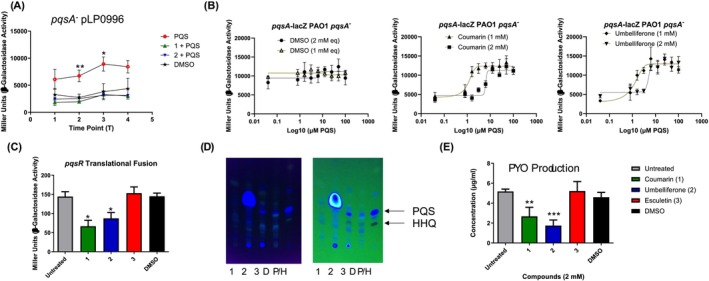
Direct QS promoter activity suppression in a *pqsA* mutant. (A) *pqsA* promoter activity in a *pqsA* mutant grown in the presence of coumarin compounds in the presence of exogenous PQS. (B) Competitive inhibition analysis of the coumarins vs. PQS PqsR interaction using a *pqsA* mutant *pqsA* promoter fusion with increasing concentrations of PQS. (C) *pqsR* promoter activity in the wild‐type *P. aeruginosa* strain in the presence of coumarin compounds. (D) TLC analysis of *P. aeruginosa* extracts from growth in the presence of coumarin compounds. Images are representative of at least three independent biological replicates. (E) PYO production and (F) second messenger signalling in *P. aeruginosa* in the presence of coumarin compounds. All data presented are the mean (±SEM) of at least three independent biological replicates. Statistical analysis was performed by one‐way ANOVA with Dunnett's post hoc corrective testing (*p ≤ 0.05, **p ≤ 0.005).

The suppression of *pqsA* promoter activity by coumarin and umbelliferone was of particular interest in light of the role played by HHQ and PQS in interkingdom communication and the host‐pathogen interaction (Reen et al. 2011). These two quorum sensing signals have previously been shown to operate through the PqsR (also known as MvfR) LysR‐Type transcriptional regulator and have exhibited interspecies and interkingdom properties in several studies (Reen et al. 2011; Maura et al. 2016; Reen, Gutiérrez‐Barranquero, et al. 2018; Kitao et al. 2018). To further explore the impact of these natural coumarin compounds on *pqsA* promoter activity, the impact of coumarins on activation of the system by exogenous PQS was explored using a *P. aeruginosa* *pqsA* mutant carrying the *pqsA*‐pLP0996 reporter. Addition of exogenous PQS led to a significant increase in *pqsA* promoter activity, thus serving as a positive control (Figure 5A). When exogenous PQS was added in the presence of coumarin compounds, coumarin and umbelliferone both blocked the activation of the *pqsA* promoter, suggesting that suppression of *pqsA* promoter activity may be the result of a direct influence on the PqsR protein (Figure 5A). To investigate this, we undertook a competitive inhibition assay, testing 2 mM concentrations of each coumarin with increasing concentrations of exogenous PQS. These assays suggest the suppression of *pqsA* promoter activity to be the result of competitive inhibition, with the dose response curves confirming the shift in suppression at lower doses of coumarin and umbelliferone (Figure 5B), though indirect effects on PQS production or upstream regulatory processes cannot be excluded. Promoter fusion analysis of a *pqsR* translational fusion indicates that *pqsR* promoter activity is itself reduced in the presence of coumarin and umbelliferone, raising the possibility that auto‐induction may be impaired, or that a secondary target for coumarins could suppress activation at this locus (Figure 5C). The suppression of *pqsA* promoter activity would be expected to result in a reduction in HHQ and PQS signal production in the presence of coumarin and umbelliferone. This was consistent with TLC analysis whereby coumarin and umbelliferone both resulted in a visible reduction in PQS/HHQ levels, while samples grown in the presence of esculetin resulted in higher signal production than the DMSO control (Figure 5D). Production of the virulence factor pyocyanin, which is regulated by the PQS system in *P. aeruginosa* , was also decreased in the samples grown with coumarin and umbelliferone when compared to the DMSO control (Figure 5E), consistent with a loss of PQS production.

### 3.5 Molecular Modelling Reveals Structural Insights Into Coumarin Species Specificity

To investigate further how coumarin (**1**) and umbelliferone (**2**) could interfere with cell–cell communication in *P. aeruginosa* , we explored the possibility that these molecules could block either the interaction between the autoinducer N‐3‐oxo‐dodecanoyl‐L‐homoserine lactone (3‐oxo‐C12‐HSL) and the transcriptional activator protein (LasR) and/or the interactions between PQS and the LysR‐type transcriptional regulator PqsR. Esculetin (**3**), which contains hydroxyl substitutions at both the 6‐ and 7‐ positions, was included as a reference point in light of its inactivity against PQS signalling.

**FIGURE 6:**
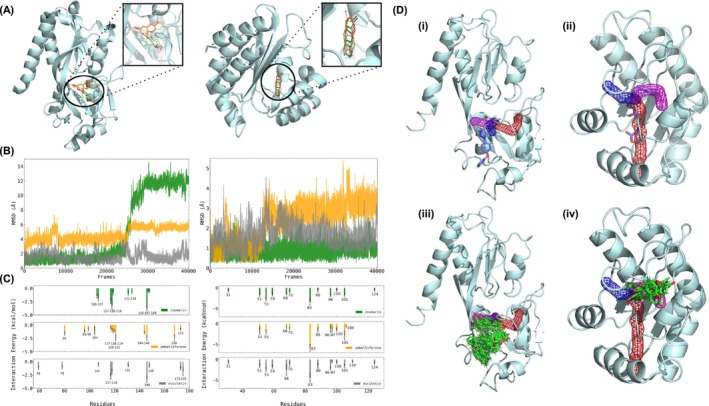
Molecular modelling of the PqsR and LasR interaction with coumarin, umbelliferone, and esculetin. (A) Representative docking poses obtained for coumarin (C‐atoms in green), umbelliferone (orange) and esculetin (grey) in PqsR (left) and in LasR (right). (B) Root‐mean square deviation (RMSD, Å) for coumarin (green line), umbelliferone (orange) and esculetin (grey) bound to PqsR (left) and LasR (right). (C) Interaction ligand‐protein energy (kcal mol −1) decompose per residue for the receptors PqsR (left) and LasR (right). Error bars count for the standard deviations (30 snapshots in a 30 ns‐window from the MD simulation). (D) Superposition of the apo structure of PqsR (i) and LasR (ii) and the crystal structure with the co‐crystallised ligand (represented as sticks in purple). In the mesh representation, tunnels 1 (blue), 2 (magenta) and 3 (red) found by the Caver are depicted. Tunnels explored by 2 during the MD simulations are presented for PqsR (iii) and LasR (iv).

First, we docked the three molecules at the binding sites found for PQS (PDB id. 6YIZ) (Schütz et al. 2021) and 3‐oxo‐C12‐HSL (PDB id. 2UV0) (Bottomley et al. 2007) in PqsR and LasR, respectively (Figure 6A). No significant differences were found between the pose solutions of the three molecules to both proteins. Subsequent Molecular Dynamics (MD) simulations revealed that while esculetin barely changes its orientation in PqsR, coumarin and umbelliferone show RMSD values above 4 Å. Much smaller RMSD variations were observed when bound to LasR (Figure 6B).

The interaction fingerprints for the three compounds bound to both receptors PqsR and LasR were computed via the algorithm MM‐ISMSA3 (Figure 6C). In general, the interactions observed in PqsR are mainly weak dispersion interactions. As an example, the C_sp_^3^H‐π interaction between the methyl groups of Ile146 in PqsR with the electron‐deficient pyrone ring of the three coumarin ligands is the one with the larger contribution. The interactions of these molecules with other aliphatic residues like Leu117 are also notable. Furthermore, the carbonyl oxygen in the pyrone ring points to the region where residues Leu106 and Ser107 are located and forms temporal hydrogen bonds with their hydroxyl and amino groups, respectively. Interestingly, the hydroxyl group on position 7‐ (umbelliferone) and on both positions 6‐ and 7‐ (esculetin) do present new hydrogen bonds for the protein‐ligand interaction. In the case of umbelliferone, a hydrogen bond is found between the hydroxyl group and the aromatic ring of Tyr168, while the carbonyl oxygen (in the pyrone ring) establishes a hydrogen bond interaction with the side chain of Gln104. In the case of esculetin, the hydroxylation on C‐6 introduces a hydrogen bond with residue Thr175, with the 7‐OH group projecting into the solvent. Regarding LasR, aromatic residues like Trp83 and Trp55 are prominent in the binding site. Indeed, Trp83 presents the largest contribution for the energy binding of the three molecules. Finally, the global binding energy (Table S2) corroborates that there are also no appreciable differences amongst the three molecules in terms of their energetic potential.

As a second strategy, we ran longer MD simulations using the apo form of the proteins embedded in an aqueous solution including 10 molecules of each of the coumarin derivatives per protein chain. This enabled us to check if the molecules in solution could enter the former binding sites explored in our docking studies. In the case of LasR we decided to use a dimer since this is the biologically relevant form as described by Bottomley and colleagues. As can be seen from the RMSD evolution along the MD simulation in Figure S6, at least one molecule of coumarin, umbelliferone and esculetin is able to approach and enter the binding site in PqsR (RMSD values highlighted with a dotted‐line box). On the other hand, none of the molecules appear to reach the active site in the case of LasR (Figure S6). In order to further explore the differential access of the ligands to the crystallographic binding site, we analysed relaxed structures of the apo form of both proteins using the software Caver (Stourac et al. 2019). In PqsR, the co‐crystallised triazolopyridine inverse agonist A overlaps mainly with the magenta and blue tunnels; in LasR, the overlap of 3‐oxo‐C12‐HSL is mainly with the red and the magenta tunnels (Figure 6D(i and iii)). Comparison of the solutions of the MD simulation for coumarin revealed that whereas in PqsR coumarin is able to explore the two tunnels where the co‐crystallised ligand is present, in LasR the corresponding one (red tunnel) is not explored (Figure 6D(ii and iv)). Calculating the size of the bottleneck radius (Å) and the average throughput for the six tunnels (Table S3), the blue tunnels were found to be the most probable for molecule transit.

### 3.6 Structure Function Profiling Indicates a Core Coumarin Framework for Species Control

**FIGURE 7:**
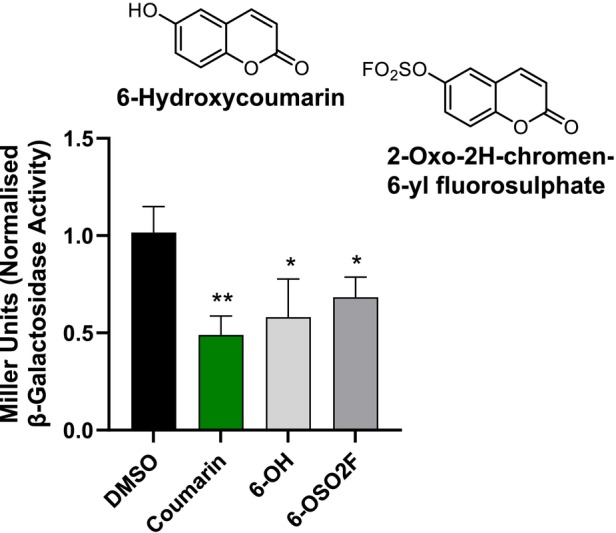
Promoter activity of *pqsA* in wild‐type PAO1 grown in the presence of coumarin, 6‐hydroxycoumarin (6‐OH) and 2‐oxo‐2H‐chromen‐6‐yl fluorosulphate (6‐OSO2F) and normalised to the untreated control. All data presented are the mean (±SEM) of at least three independent biological replicates. Statistical analysis was performed by one‐way ANOVA with Dunnett's post hoc corrective testing (*p ≤ 0.05).

Evidence of structural differences underpinning the biological activity of the coumarin compounds was apparent in the phenotypic studies with hydroxylation (comparing compounds **1–3**) and the hydroxyl vs. methoxy group at position 4 (comparing compounds **5–6**) resulting in markedly different phenotypes in susceptible pathogens. We therefore investigated hydroxylation at the 6‐position, reasoning that (i) either the presence of the 6‐OH was the cause of esculetin not interfering with PqsR activity, or (ii) that the inhibitory effect could tolerate either 6‐OH or 7‐OH, but not both. Favouring the latter hypothesis, 6‐hydroxycoumarin was found to inhibit *pqsA* promoter activity, comparable to coumarin (Figure 7). To determine if a different functionality to the OH group could be tolerated at this position, we synthesised fluorosulphate substituted analogues of 6‐hydroxycoumarin (6‐OH, **File**) and umbelliferone (7‐OH). While issues with solubility prohibited us from studying the 7‐OH analogue at the required concentration, the 6‐OH analogue (2‐oxo‐2H‐chromen‐6‐yl fluorosulphate) retained antagonistic activity towards the *pqsA* promoter activity, suggesting that the mechanism of inhibition could tolerate more than an ‐OH substitution (Figure 7).

To explore the structure–function relationship in a broader sense across the coumarin framework, a total of four coumarin analogues (**7**–**10**) were tested for their impact on biofilm and virulence. In addition to warfarin (**7**) and a fluorescent probe (**8**), two analogues of the pyrone component of the coumarin structure (compounds **9–10**) were included in this analysis. With the exception of warfarin leading to a slower growth rate and lower final biomass in *S. aureus* NCDO949, growth was uninhibited by the coumarin analogues (Figure S7).

**FIGURE 8:**
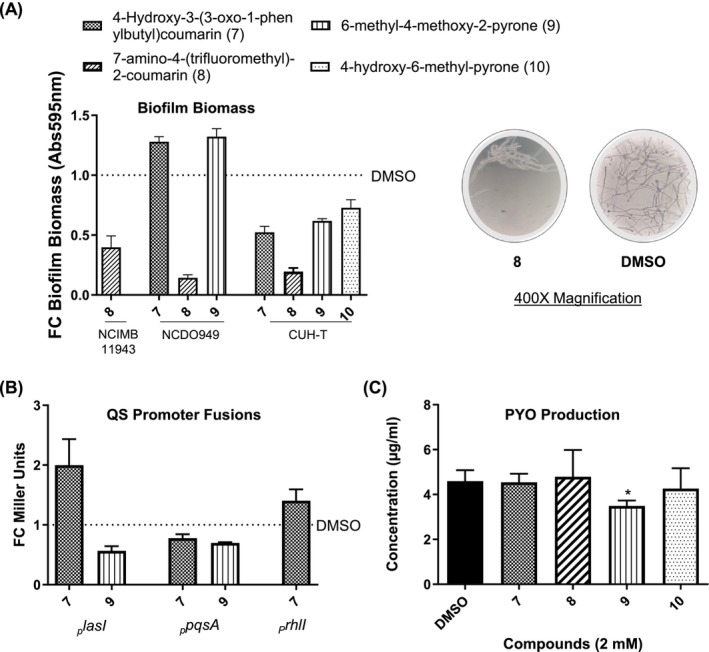
Profile of activity of synthetic coumarin compounds against key pathogens. (A) Fold change in biofilm formation and visualisation of *A. fumigatus* Af293 hyphal structures (data presented is representative of multiple micrographs taken across three independent biological replicates); (B) Fold change in promoter activity for QS systems; (C) PYO production in *P. aeruginosa* PAO1. All data presented is the mean (±SEM) of at least three independent biological replicates.

None of the synthetic analogues exhibited suppression of pigmentation in the *C. violaceum* DSM30191 or *Serratia* sp. SP15 biosensors (Figures S8 and S9). While none of the modified coumarin compounds exhibited anti‐biofilm activity against *P. aeruginosa* , compound **8** was notable for its biofilm inhibitory activity towards *A. fumigatus* , *Staphylococcus* and *E. coli* , while **9** was also suppressive of biofilm formation in these species with the exception of *E. coli* (Figure 8A and Figures S10–S12). It was noteworthy that while **7** and **9** significantly enhanced biofilm formation in *S. aureus* NCDO949, both had a strong suppressive effect against *S. haemolyticus* CUH‐T. From the perspective of the three QS systems in *P. aeruginosa* PAO1, compound **7** led to increased *lasI* promoter activity, while **9** resulted in reduced *lasI* and *pqsA* activity (Figure 8B and Figure S13). Compounds **8** and **10** did not influence promoter fusion activity (Figure 8B and Figure S13). It was also notable that compound **9** led to a reduction in PYO production in *P. aeruginosa* (Figure 8C).

## 4 Discussion

The increasing prevalence of antibiotic resistance, especially in nosocomial infections, is becoming a serious global threat to public safety (Chokshi et al. 2019). Apart from the well documented projected increased mortality arising from AMR, illnesses due to antibiotic resistant bacteria may also take longer to resolve and will increase healthcare expenses (Costelloe et al. 2010). The dearth of antibiotics in development has led to studies on alternative treatment strategies, such as antimicrobial molecules and compounds that can target specific virulence factors and communication systems, that is, quorum sensing (Reen, Phelan, Gallagher, et al. 2016; Reen, Phelan, Woods, et al. 2016; Whiteley et al. 2017; Reen, McGlacken, and O'Gara 2018; Soukarieh et al. 2018; Ó Muimhneacháin et al. 2018; Linciano et al. 2020). Though the view was long held that targeting of QS poses a lower risk of resistance developing, there is growing evidence that resistance has been encountered in laboratory and clinical strains (Maeda et al. 2012; Kalia et al. 2014; Grossman et al. 2020; Patel et al. 2025). While it is likely that a less potent ‘Darwinian’ selection may manifest in the presence of these interventions, the design and future development of small molecular approaches to pathogen control must heed these concerns.

Aside from their emerging role in shaping the rhizosphere, or perhaps as a consequence of this, coumarins present a significant opportunity as a framework for the design of therapeutic interventions against opportunistic pathogens. The coumarin family of compounds have shown attractive pharmacological characteristics such as anti QS, antibiofilm, antifungal and antioxidant properties (Ojima et al. 2016; Slobodníková et al. 2016; Pan et al. 2017; Reen, Gutiérrez‐Barranquero, et al. 2018). Coumarins have also demonstrated repression of the classic AHL, and the more species‐restricted alkyl‐hydroxyquinolone (AHQ) quorum sensing systems (Gutiérrez‐Barranquero et al. 2015; Reen, Gutiérrez‐Barranquero, et al. 2018; Zhang et al. 2018; Qais et al. 2021; Tajani et al. 2023). The importance of the hydroxyl group with respect to QS inhibition uncovered in this study is consistent with previous findings by D'Almeida and colleagues where coumarin molecules with hydroxyl groups on the aromatic ring were found to display enhanced inhibition of biofilm formation by *P. aeruginosa* when compared with coumarins with substituents in positions 3 and 4 or without the double 3,4 bond (D'Almeida et al. 2017). The degree of hydroxylation and/or position of the hydroxy groups has an impact on the anti‐infective activity, though substitution of the ‐OH in the natural coumarin compounds with a fluorosulphate group would suggest that the ‐OH is not absolutely required (Figure 7), at least for PqsR inhibition. QS and biofilm formation were inhibited by coumarin (no hydroxy group), umbelliferone (7‐ hydroxy) and esculetin (6,7‐ dihydroxy) in several pathogenic species. Esculetin exhibited the broadest spectrum of activity, targeting *P. aeruginosa* , *S. haemolyticus* , *A. fumigatus* and *S. aureus* , enhancing biofilm in the latter (Figures 1 and 2). Interestingly, while the early stages of biofilm were affected, attachment itself was unchanged upon addition of either of the three compounds. (Figure 3). Both coumarin and umbelliferone altered EPS production, suppressed *pqsA* transcriptional and *pqsR* translational activity, and PYO production in *P. aeruginosa* (Figures 3, 4, 5). Esculetin on the other hand did not influence these phenotypes, suggesting a specific interaction related to the hydroxylation (or lack thereof) at positions 6‐ and 7‐ (Figures 3, 4, 5). It is worth noting here that the EPS analysis performed is largely descriptive and insufficient to conclusively establish that coumarins exert their antibiofilm effect through specific modulation of exopolysaccharide production. Further quantitative, compositional, and microscopic analysis will be required before a direct role or correlation can be established.

Molecular modelling of the coumarin, umbelliferone, and esculetin interaction with the LasR and PqsR receptor proteins did not differentiate their binding affinities or interaction modalities (Figure 6). Overall, our computational data suggest that the coumarin derivatives may be able to bind to both PsqR and LasR proteins, but with no significant differences for their binding mode. However, it is worth noting that whereas these coumarin derivatives access the explored binding site at PqsR, access to the binding site of 3‐oxo‐C12‐HSL in LasR is not evident in the ns‐time scale of MD simulations. It is also important to acknowledge here that the molecular modelling does not provide direct mechanistic evidence of receptor targeting and that binding affinity assays will be required to conclusively establish the nature of the coumarin‐PqsR and coumarin‐LasR relationships. This will form the basis of future mechanistic studies. Furthermore, while the consequence of PqsR suppression was evidenced in reduced HHQ/PQS and PYO production, the impact of reduced *pqsE* expression must also be considered in future studies (Borgert et al. 2022).

Some interesting insights also emerge from comparison of the coumarin structures. 4‐hydroxy‐2‐coumarin (**5**) exhibited similarities in biological activity to esculetin, enhancing biofilm formation in *S. aureus* NCDO949 and inhibiting biofilm formation in *S. haemolyticus* CUH‐T (Figure 2). The position of the hydroxy group (4‐hydroxy) and its proximity to the 6‐hydroxy group may be relevant here. A methoxy group at the 4‐position (4‐methoxy‐2‐coumarin) seems to be well tolerated (compounds **6** and **9**) and resulted in strong inhibition of *A. fumigatus* Af293 biofilm formation. Conversely, a hydroxyl group at the same position appears to abrogate activity (compounds **5** and **10**). 7‐amino‐4‐(trifluoromethyl)‐2‐coumarin (compound **8**) was a strong inhibitor of *Staphylococcus* species biofilm formation, being also active against *A. fumigatus* Af293. Fine tuning of signal production in bacterial pathogens may offer a more precise approach to infection control. It should be noted, however, that the species tested here do not share the same QS signals or mechanisms of action, notwithstanding the finding that they can be affected by the same small molecule(s). We consider two possibilities here (though there may be more): (i) there is an unidentified receptor or response that is common to each one through which these molecules operate or (ii) there are distinct mechanisms independent of QS through which biofilm and other phenotypes are affected. Structural insights at the molecular level should also be viewed in context of the recently reported diversification of the PqsR protein, being amongst the most variable of the LTTR proteins encoded in *P. aeruginosa* (Deery et al. 2024). Further mechanistic insights will be required to establish which (if either) of these explains the spectrum of coumarin activity.

The evidence from this study would indicate that coumarins and structurally similar compounds can be either ‘bad communicators’ or ‘coercives’ in nature, depending on dose and the molecules‐species in question. ‘Bad communicators’ can be considered to have no direct implication on the integrity of the receiver cells, they are not harming the cells but are scrambling the communication that is, the QSI molecules. ‘Coercive’ molecules negatively impact the receiver cells as is the case of bactericidal antibiotics which prevents the cells achieving minimal signal threshold (Diggle et al. 2007). The data presented here would suggest that the anti‐virulence activities of coumarins occurs largely as a consequence of growth‐independent alterations to cell behaviour. In some ways, it could be viewed in the same light as the antimicrobial as a weapon or signal (Linares et al. 2006; Fajardo et al. 2009; Granato et al. 2019); dose is key to contextualising the outcome. When one considers the distinct coumarin structures exhibiting activity against pathogens that independently carry the LuxIR‐AHL or LuxS or AIP systems, it could be reasoned that the anti‐infective activity of coumarins will be underpinned by interactions outside of these classical systems. It is important that these complexities are deciphered, particularly where coumarins are emerging as significant signals within the complex polymicrobial dynamics of natural communities.

The production of coumarin compounds by a range of plant species may also offer a novel mechanism for control of opportunistic pathogens, whereby the profile of coumarin compounds introduced by a plant‐rich diet may lead to moderation of pathogenic species within communities; the dynamics of the rhizosphere introduced into a host‐context (McCarthy and O'Gara 2015). While this would mean that coumarin compounds would need to remain stable during passage through the host, and would need to be refractive to biotransformation, it is worth exploring the possibility that coumarin compounds could themselves act as behavioural modulators in human pathogenic species, both fungal and bacterial. Some evidence of a role for the gut microbiome in biotransformation of coumarins has already been reported (Theilmann et al. 2017). However, a greater understanding of the ecology of microbial communities and the nature of the small molecule interactions governing their dynamics at a polymicrobial or community level is warranted, such that better and more precise interventions can be designed.

## Author Contributions

**David F. Woods:** investigation, writing – review and editing. **Dylan Boon:** investigation, writing – original draft, writing – review and editing. **Muireann Carmody:** investigation, funding acquisition, writing – original draft, writing – review and editing. **F. Jerry Reen:** conceptualization, investigation, funding acquisition, writing – original draft, writing – review and editing, formal analysis, supervision, resources. **Pedro A. Sánchez‐Murcia:** funding acquisition, investigation, writing – original draft, writing – review and editing, formal analysis, supervision, resources. **Gerard P. McGlacken:** investigation, funding acquisition, writing – review and editing, formal analysis, supervision, resources. **Benjamin O'Rourke:** investigation, writing – original draft, writing – review and editing. **Daniel Platero‐Rochart:** investigation, writing – original draft, writing – review and editing. **Antje Gloe:** investigation, writing – review and editing.

## Funding

This work was supported by Science Foundation Ireland (12/RC/2275_2, SFI/12/IP/1315, 21/FFP‐A/8784, SFI 15/RI/3221, 21/RI/9705), Health Research Board (ILP‐POR‐2019‐004), Irish Research Council for Science, Engineering and Technology (GOIPG/2021/692), Medizinische Universität Graz, MedBioNode.

## Conflicts of Interest

The authors declare no conflicts of interest.

## Supporting information


**Table S1:** Strains and plasmids used in this study.
**Table S2:** Total ligand‐protein interaction energy (kcal mol −1) obtained using MM‐ISMSA3 (values are reported as total energy ± standard deviation). A 30 ns‐ window with a total of 30 snapshots were used in each case for the analysis.
**Table S3:** Bottleneck radius (°A) and average throughput for the tunnels found by Caver web server.
**Figure S1:** AHL‐mediated quorum sensing inhibition. Visualisation of quorum sensing inhibition (A) in the absence and (B) presence of XTT for detection of metabolic activity. Coumarin compounds are added at 50 (bottom left), 100 (top left) and 150 (top right) to each well. All data presented is representative of three independent biological replicates. PBS is presented as a control.
**Figure S2:** Dose dependent biofilm inhibition in the presence of natural coumarin compounds. (A) Summary inhibition of biofilm formation by coumarin compounds at 0.01, 0.1, 1 and 2 mM. (B) Inhibition of biofilm formation by 1 mM coumarin compounds. Each dataset is the mean (±SEM) of at least three independent biological replicates. Statistical analysis was performed by one‐way ANOVA with Dunnett's post hoc corrective testing (*p ≤ 0.05).
**Figure S3:** MIC analysis of *P. aeruginosa* biofilm formation in the presence of coumarin, umbelliferone, and esculetin. Data presented is the mean (±SEM) of three independent biological replicates.
**Figure S4:** EPS production in *P. aeruginosa* PA14 in the presence of coumarin compounds. Colony formation was monitored over 120 h and visualised for evidence of EPS production. DMSO was included as a carrier control, with the constitutive EPS producing PA14 TnM *tbpA*‐D mutant included for comparison.
**Figure S5:** Growth kinetics in the presence of 2 mM coumarin compounds. Test organisms were assayed on a Bioscreen C plate reader. All data presented is the mean (±SEM) of three independent biological replicates.
**Figure S6:** Molecular modelling. (A) RMSD (Å) of the 10 small molecules with respect to docking results in the PqsR receptor. Highlighted we show the molecules with high resemblance with the docking results. (B) RMSD (Å) of the 20 small molecules with respect to docking results in the LasR receptor. Each row of images corresponds to one chain of the dimer.
**Figure S7:** Growth kinetics in the presence of 2 mM coumarin analogues. Test organisms were assayed on a Bioscreen C plate reader. All data presented is the mean (±SEM) of three independent biological replicates.
**Figure S8:** Viable cell count growth analysis. The effect on growth of (A) CUH‐T and (B) NCDO949 in the presence of compound 8 at 2 mM. Due to the incompatibility of using compound 8 with the Bioscreen, the growth was measured by viable cell count plating over a 12 h period and is expressed as cfu/ml. All data presented is the mean (±SEM) of three independent biological replicates.
**Figure S9:** Biosensor analysis of coumarin analogue structures indicates lack of QS suppression upon modification of the core coumarin. (i) zone inhibition of pigment production (ii‐iii) visualisation of pigment production in biosensor strains.
**Figure S10:** Biofilm formation in the presence of coumarin analogues. Biofilm formation is presented as OD_595nm_. Each dataset is the mean (±SEM) or is representative (images) of at least three independent biological replicates. Statistical analysis was performed by one‐way ANOVA with Dunnett's post hoc corrective testing (* *p* ≤ 0.05, ** *p* ≤ 0.005, *** *p* ≤ 0.001).
**Figure S11:** Dose dependent biofilm formation in the presence of coumarin analogues. Each dataset is the mean (±SEM) of at least three independent biological replicates. Statistical analysis was performed by one‐way ANOVA with Dunnett's post hoc corrective testing (* *p* ≤ 0.05, ** *p* ≤ 0.005, *** *p* ≤ 0.001).
**Figure S12:** EPS production in *P. aeruginosa* PA14 in the presence of synthetic coumarin compounds. All data presented is representative of three independent biological replicates.
**Figure S13:** Quorum sensing promoter fusion analysis in *P. aeruginosa*. (A‐C) The three systems operating in *P. aeruginosa* were studied in the presence of synthetic coumarin compounds. All data presented is the mean (±SEM) of at least three independent biological replicates. (D–F) Analysis of promoter activity in response to compound 8 was performed using viable cell counts rather than OD_600nm_.
**Methods File S1:** Experimental Methods: Computational.
**Methods File S2:** Experimental Methods: Chemistry.

## Data Availability

All data pertaining to the manuscript is contained within the Supporting Information, and any additional materials described in the manuscript, including all relevant raw data, will be made freely available to any researcher wishing to use them for non‐commercial purposes.
