# Supplementary material for: Coumarins Disrupt Cell–Cell Communication and Virulence in Priority Pathogens: Targeting the PQS Signalling System in Pseudomonas aeruginosa
Source: Microb Biotechnol. 2026 Jun 26;19(7):e70404. doi: 10.1111/1751-7915.70404 (PMC13307335; doi:10.1111/1751-7915.70404)
Supplement: Supplementary file 1 — Table S1: Strains and plasmids used in this study. Table S2: Total ligand‐protein interaction energy (kcal mol −1) obtained using MM‐ISMSA3 (values are reported as total energy ± standard deviation). A 30 ns‐ window with a total of 30 snapshots were used in each case for the analysis. Table S3: Bottleneck radius (°A) and average throughput for the tunnels found by Caver web server. Figure S1: AHL‐mediated quorum sensing inhibition. Visualisation of quorum sensing inhibition (A) in the absence and (B) presence of XTT for detection of metabolic activity. Coumarin compounds are added at 50 (bottom left), 100 (top left) and 150 (top right) to each well. All data presented is representative of three independent biological replicates. PBS is presented as a control. Figure S2: Dose dependent biofilm inhibition in the presence of natural coumarin compounds. (A) Summary inhibition of biofilm formation by coumarin compounds at 0.01, 0.1, 1 and 2 mM. (B) Inhibition of biofilm formation by 1 mM coumarin compounds. Each dataset is the mean (±SEM) of at least three independent biological replicates. Statistical analysis was performed by one‐way ANOVA with Dunnett's post hoc corrective testing (*p ≤ 0.05). Figure S3: MIC analysis of P. aeruginosa biofilm formation in the presence of coumarin, umbelliferone, and esculetin. Data presented is the mean (±SEM) of three independent biological replicates. Figure S4: EPS production in P. aeruginosa PA14 in the presence of coumarin compounds. Colony formation was monitored over 120 h and visualised for evidence of EPS production. DMSO was included as a carrier control, with the constitutive EPS producing PA14 TnM tbpA‐D mutant included for comparison. Figure S5: Growth kinetics in the presence of 2 mM coumarin compounds. Test organisms were assayed on a Bioscreen C plate reader. All data presented is the mean (±SEM) of three independent biological replicates. Figure S6: Molecular modelling. (A) RMSD (Å) of the 10 small molecules with respe [file MBT2-19-e70404-s001.pdf]

Research Article Supplementary File Legends

Coumarins disrupt cell-cell communication and virulence in priority pathogens, targeting the PQS signalling system in *Pseudomonas aeruginosa*

Dylan Boon[1¥~], Benjamin O'Rourke[1¥], Muireann Carmody[1,2], David F. Woods[1], Antje Gloe[1,3], Daniel Platero-Rochart[4], Pedro A. Sánchez-Murcia[4], Gerard P. McGlacken[2,5] and F. Jerry Reen[1,5*]

Supplementary Table S1. Strains and plasmids used in this study.

Supplementary Table S2: Total ligand-protein interaction energy (kcal mol −1) obtained using MM-ISMSA3 (values are reported as total energy ± standard deviation). A 30ns-window with a total of 30 snapshots were used in each case for the analysis.

Supplementary Table S3: Bottleneck radius (˚A) and average throughput for the tunnels found by Caver web server.

Supplementary Figure S1. AHL-mediated quorum sensing inhibition. Visualisation of quorum sensing inhibition (**A**) in the absence and (**B**) presence of XTT for detection of metabolic activity. Coumarin compounds are added at 50 (bottom left), 100 (top left), and 150 (top right) to each well. All data presented is representative of three independent biological replicates. PBS is presented as a control.

Supplemental Figure S2. Dose dependent biofilm inhibition in the presence of natural coumarin compounds. (**A**) Summary inhibition of biofilm formation by coumarin compounds at 0.01, 0.1, 1, and 2 mM. (**B**) Inhibition of biofilm formation by 1 mM coumarin compounds. Each dataset is the mean (+/- SEM) of at least three independent biological

replicates. Statistical analysis was performed by one-way ANOVA with Dunnett's post-hoc corrective testing (* $p \leq 0.05$).

Supplementary Figure S3. MIC analysis of *P. aeruginosa* biofilm formation in the presence of coumarin, umbelliferone, and esculetin. Data presented is the mean (+/- SEM) of three independent biological replicates.

Supplementary Figure S4. EPS production in *P. aeruginosa* PA14 in the presence of coumarin compounds. Colony formation was monitored over 120 hrs and visualised for evidence of EPS production. DMSO was included as a carrier control, with the constitutive EPS producing PA14 TnM *tbpA-D* mutant included for comparison.

Supplementary Figure S5. Growth kinetics in the presence of 2 mM coumarin compounds. Test organisms were assayed on a Bioscreen C plate reader. All data presented is the mean (+/- SEM) of three independent biological replicates.

Supplementary Figure S6. Molecular modelling. (A) RMSD (Å) of the 10 small molecules with respect to docking results in the PqsR receptor. Highlighted we show the molecules with high resemblance with the docking results. **(B)** RMSD (Å) of the 20 small molecules with respect to docking results in the LasR receptor. Each row of images corresponds to one chain of the dimer.

Supplementary Figure S7. Growth kinetics in the presence of 2 mM coumarin analogues. Test organisms were assayed on a Bioscreen C plate reader. All data presented is the mean (+/- SEM) of three independent biological replicates.

Supplementary Figure S8. Viable cell count growth analysis. The effect on growth of (**A**) CUH-T and (**B**) NCDO949 in the presence of compound 8 at 2 mM. Due to the incompatibility of using compound 8 with the Bioscreen, the growth was measured by viable

cell count plating over a 12 hr period and is expressed as cfu/ml. All data presented is the mean (+/- SEM) of three independent biological replicates.

Supplementary Figure S9. Biosensor analysis of coumarin analogue structures indicates lack of QS suppression upon modification of the core coumarin. (i) zone inhibition of pigment production (ii-iii) visualisation of pigment production in biosensor strains.

Supplemental Figure S10. Biofilm formation in the presence of coumarin analogues. Biofilm formation is presented as OD_{595nm}. Each dataset is the mean (+/- SEM) or is representative (images) of at least three independent biological replicates. Statistical analysis was performed by one-way ANOVA with Dunnett's post-hoc corrective testing (* $p \leq 0.05$, ** $p \leq 0.005$, *** $p \leq 0.001$).

Supplemental Figure S11. Dose dependent biofilm formation in the presence of coumarin analogues. Each dataset is the mean (+/- SEM) of at least three independent biological replicates. Statistical analysis was performed by one-way ANOVA with Dunnett's post-hoc corrective testing (* $p \leq 0.05$, ** $p \leq 0.005$, *** $p \leq 0.001$).

Supplementary Figure S12. EPS production in *P. aeruginosa* PA14 in the presence of synthetic coumarin compounds. All data presented is representative of three independent biological replicates.

Supplementary Figure S13. Quorum sensing promoter fusion analysis in *P. aeruginosa*. **(A-C)** The three systems operating in *P. aeruginosa* were studied in the presence of synthetic coumarin compounds. All data presented is the mean (+/- SEM) of at least three independent biological replicates. **(D-F)** Analysis of promoter activity in response to compound **8** was performed using viable cell counts rather than OD_{600nm}.

Supplementary Methods File S1. Experimental Methods: Computational.
Supplementary Methods File S2. Experimental Methods: Chemistry

Table S1. Strains and plasmids used in this study

Strain/plasmid	Description	Reference/source
PA14	*P. aeruginosa* burn wound isolate, Pennsylvania USA	Mathee K., 2018
PAO1	*P. aeruginosa* wound isolate, Australia	Holloway B.W., 1955
NCDO949	*S. aureus* type strain, isolated from human pleural fluid	NCTC, Shinfield UK
CUH-T	*S. haemolyticus* clinical isolate, Ireland	UCC Collection
NCIMB11943	*E. coli* type strain, isolated from urine of cystitis patient	NCIMB, Scotland
PAO1 p*pqsA*	PAO1 containing the pLP0996 plasmid; Cb^R	McGrath *et al*., 2004
PAO1 p*lasI*	PAO1 containing the *lasI*-pMP220 plasmid; Tc^R	Baysse *et al*., 2005
PAO1 p*rhlI*	PAO1 containing the *rhlI*-pMP220 plasmid; Tc^R	Baysse *et al*., 2005
PA14 *tbpA*	PA14 with mariner transposon insertion in PA14_13660 (*tbpA*); Gm^R	Liberati *et al*., 2006

Supplementary Table S2: Total ligand-protein interaction energy (kcal mol −1) obtained using MM-ISMSA3 (values are reported as total energy ± standard deviation). A 30ns-window with a total of 30 snapshots were used in each case for the analysis.

Energy (kcal mol^{-1})

Ligand/Receptor	PqsR	LasR
Coumarin (**1**)	-20.61 ± 1.81	-21.75 ± 1.37
Umbelliferone (**2**)	-20.14 ± 1.60	-25.77 ± 1.96
Esculetin (**3**)	-19.53 ± 1.66	-26.48 ± 1.70

Supplementary Table S3: Bottleneck radius (˚A) and average throughput for the tunnels found by Caver web server.

Tunnel	Bottleneck Radius (*A*)	Avg. Throughput
**PqsR**		
1 (blue)	1.74	0.82
2 (green)	1.04	0.68
3 (red)	1.04	0.58
**LasR**		
1 (blue)	1.41	0.74
2 (green)	0.94	0.56
3 (red)	1.13	0.49

(A)

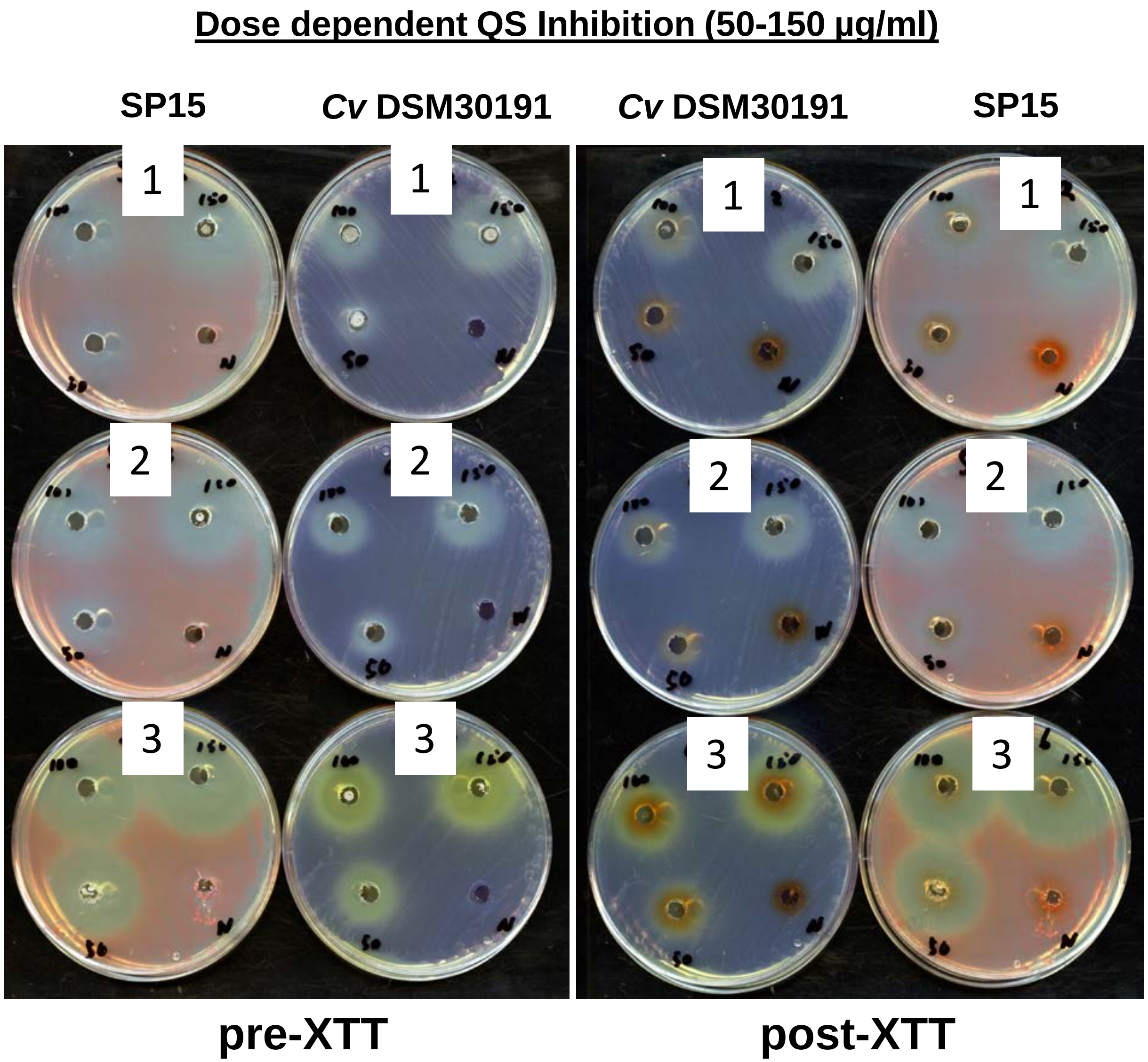
Dose dependent QS Inhibition (50-150 µg/ml)
SP15
Cv DSM30191
Cv DSM30191
SP15
1
1
1
1
2
2
2
2
3
3
3
3
pre-XTT
post-XTT

Figure S1

Figure S2

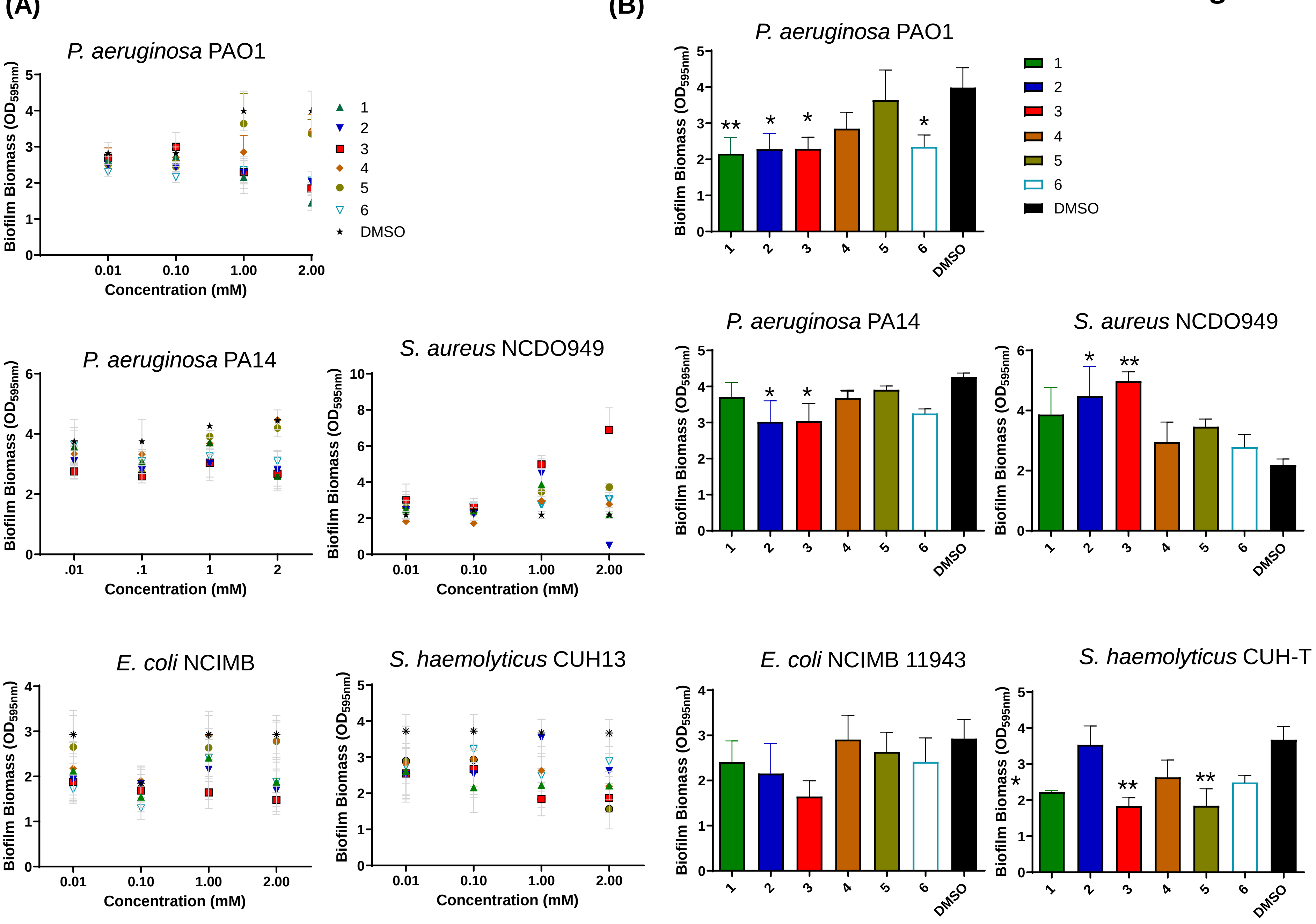
(A)
P. aeruginosa PAO1
Biofilm Biomass (OD595nm)
Concentration (mM)
1
2
3
4
5
6
DMSO
P. aeruginosa PA14
S. aureus NCDO949
E. coli NCIMB
S. haemolyticus CUH13
(B)
P. aeruginosa PAO1
P. aeruginosa PA14
S. aureus NCDO949
E. coli NCIMB 11943
S. haemolyticus CUH-T

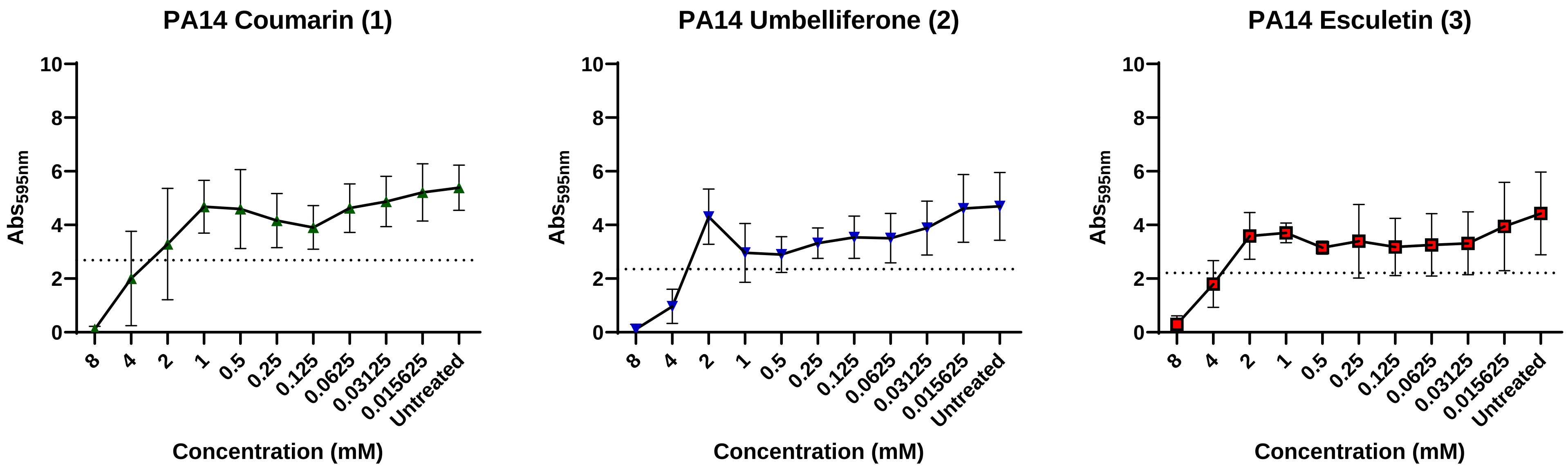
PA14 Coumarin (1)
PA14 Umbelliferone (2)
PA14 Esculetin (3)
Abs595nm
0
2
4
6
8
10
8
4
2
1
0.5
0.25
0.125
0.0625
0.03125
0.015625
Untreated
Concentration (mM)

Figure S3

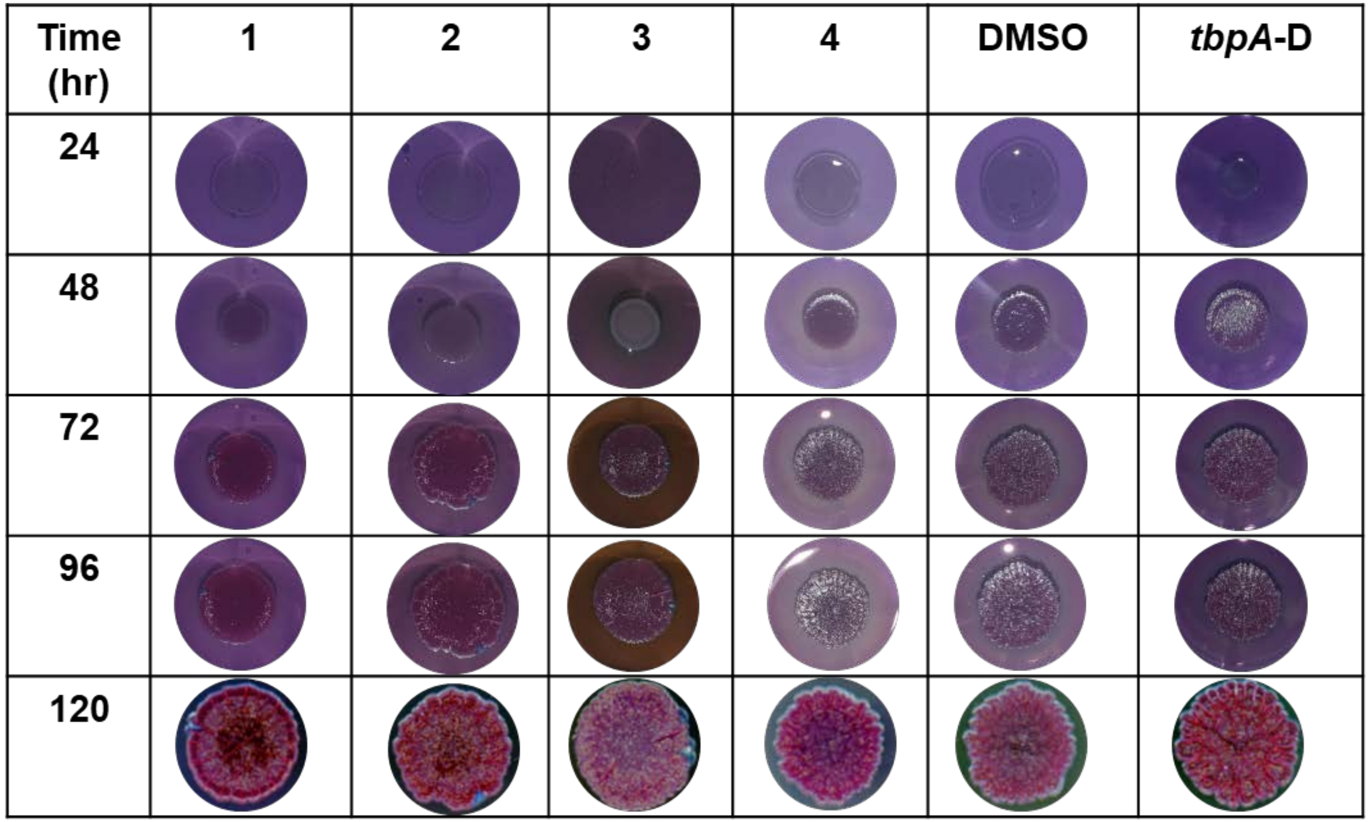

Figure S4

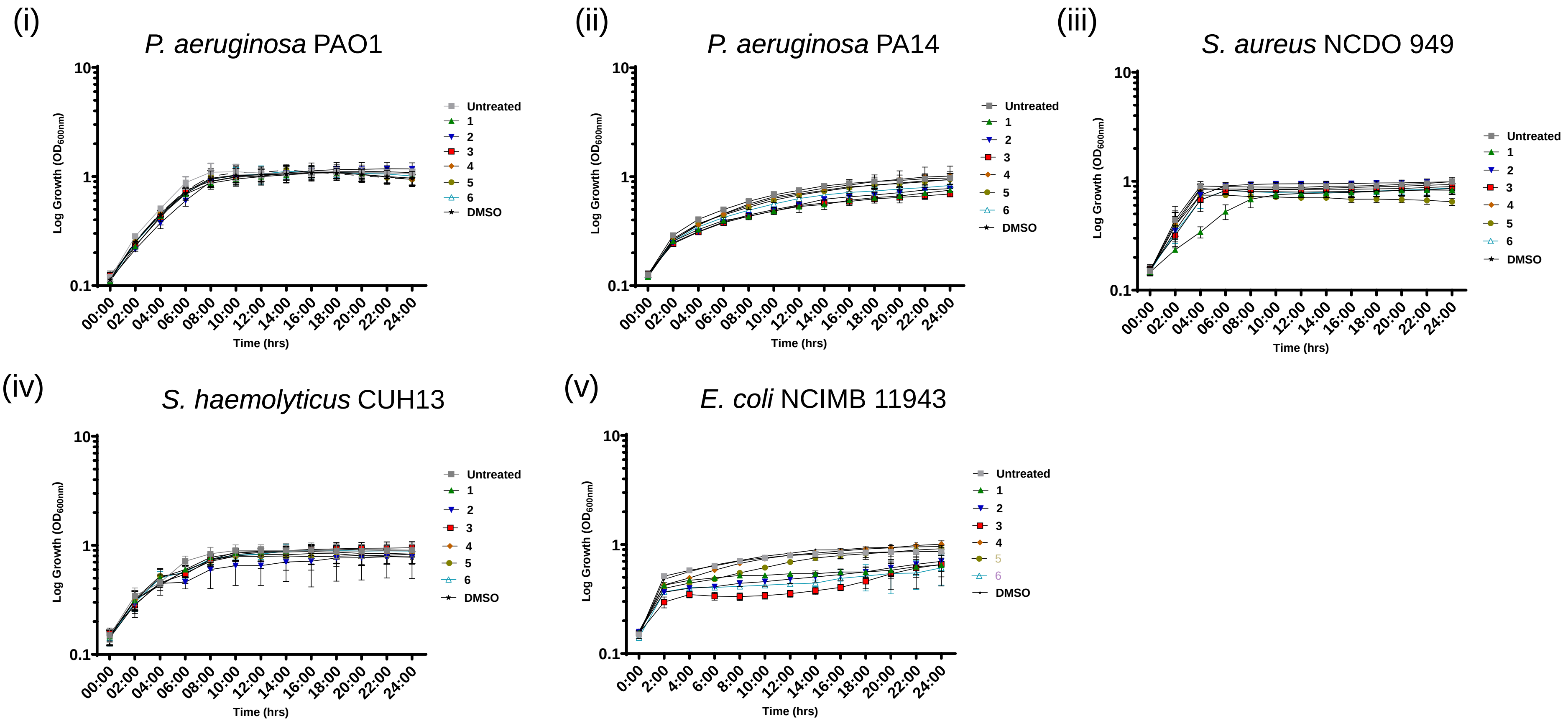
(i)
P. aeruginosa PAO1
(ii)
P. aeruginosa PA14
(iii)
S. aureus NCDO 949
(iv)
S. haemolyticus CUH13
(v)
E. coli NCIMB 11943
Log Growth (OD600nm)
10
1
0.1
Time (hrs)
00:00
02:00
04:00
06:00
08:00
10:00
12:00
14:00
16:00
18:00
20:00
22:00
24:00
0:00
2:00
4:00
6:00
8:00
Untreated
1
2
3
4
5
6
DMSO

Figure S5

(A)

(B)

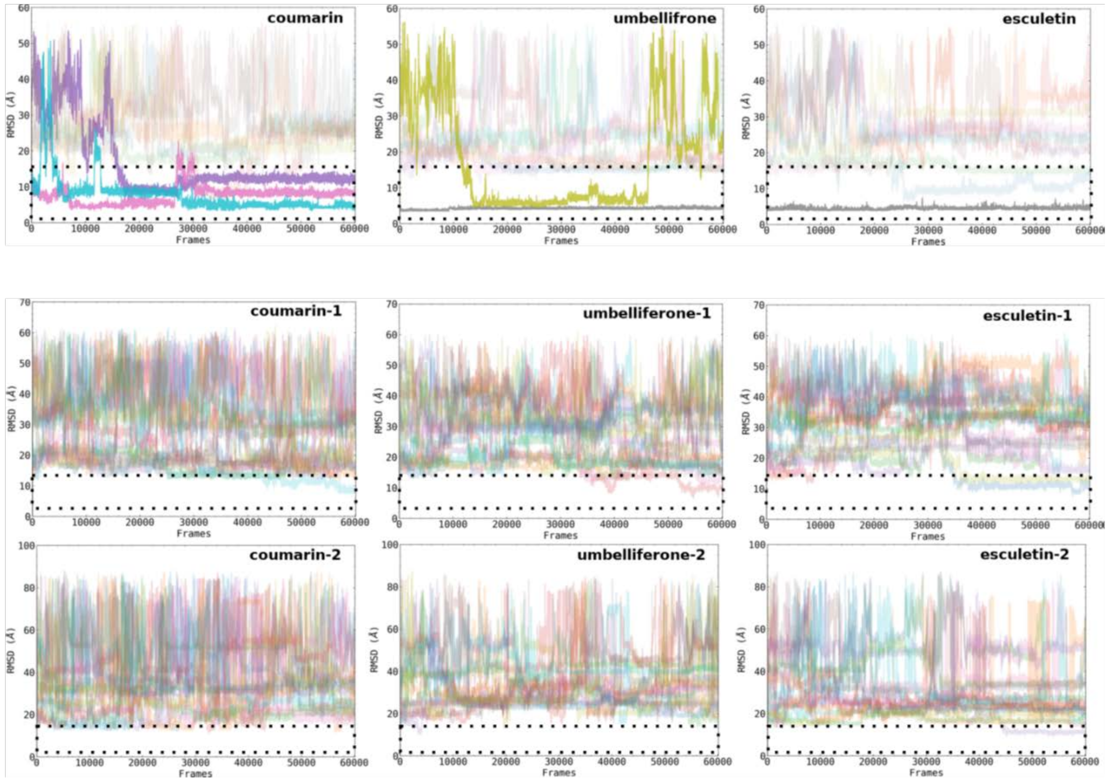
coumarin
umbellifrone
esculetin
coumarin-1
umbelliferone-1
esculetin-1
coumarin-2
umbelliferone-2
esculetin-2
RMSD (Å)
Frames

Figure S6

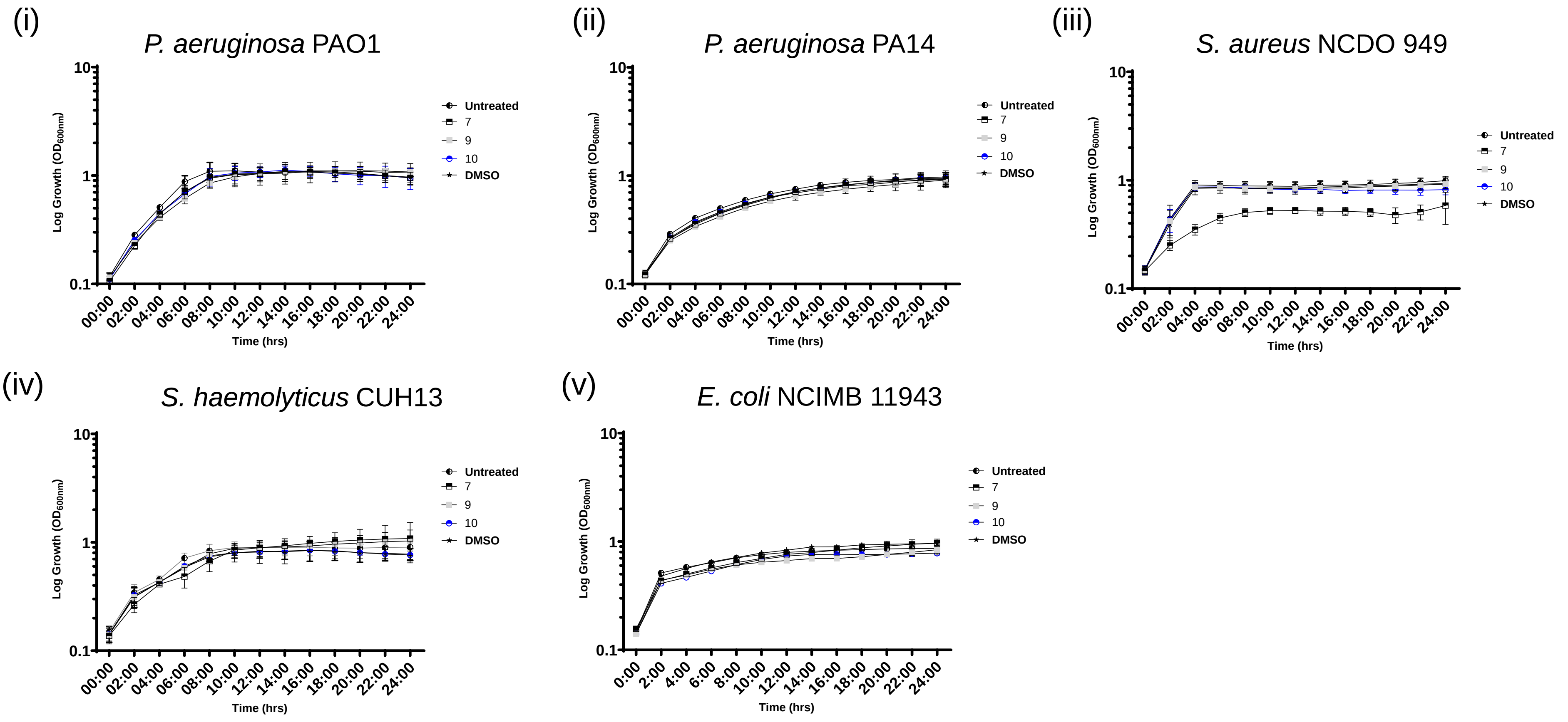
(i)
P. aeruginosa PAO1
(ii)
P. aeruginosa PA14
(iii)
S. aureus NCDO 949
(iv)
S. haemolyticus CUH13
(v)
E. coli NCIMB 11943
Log Growth (OD600nm)
Time (hrs)
Untreated
7
9
10
DMSO
10
1
0.1
00:00
02:00
04:00
06:00
08:00
10:00
12:00
14:00
16:00
18:00
20:00
22:00
24:00
0:00
2:00
4:00
6:00
8:00

Figure S7

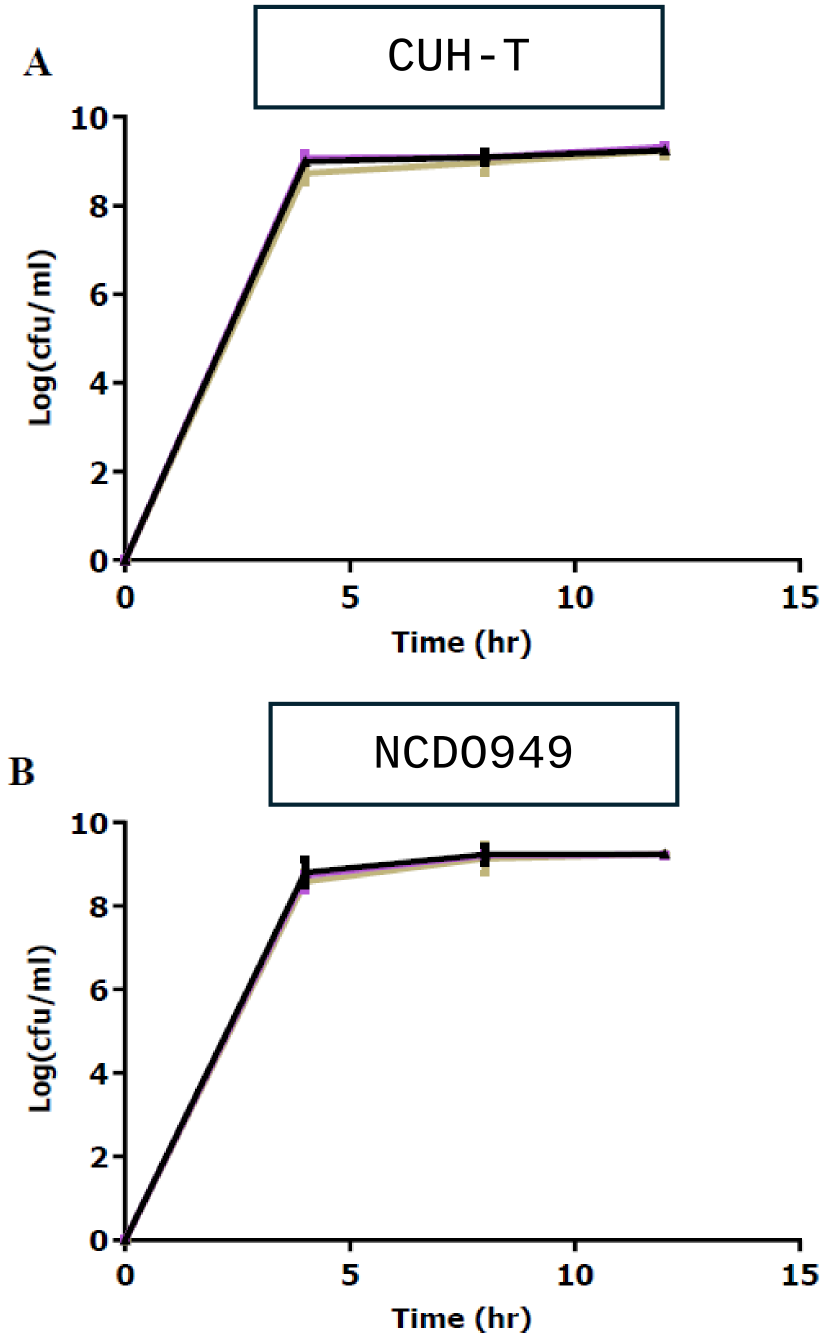
A
CUH-T
10
8
6
4
2
0
Log(cfu/ml)
0
5
10
15
Time (hr)
B
NCDO949
10
8
6
4
2
0
Log(cfu/ml)
0
5
10
15
Time (hr)

Figure S8

(A)

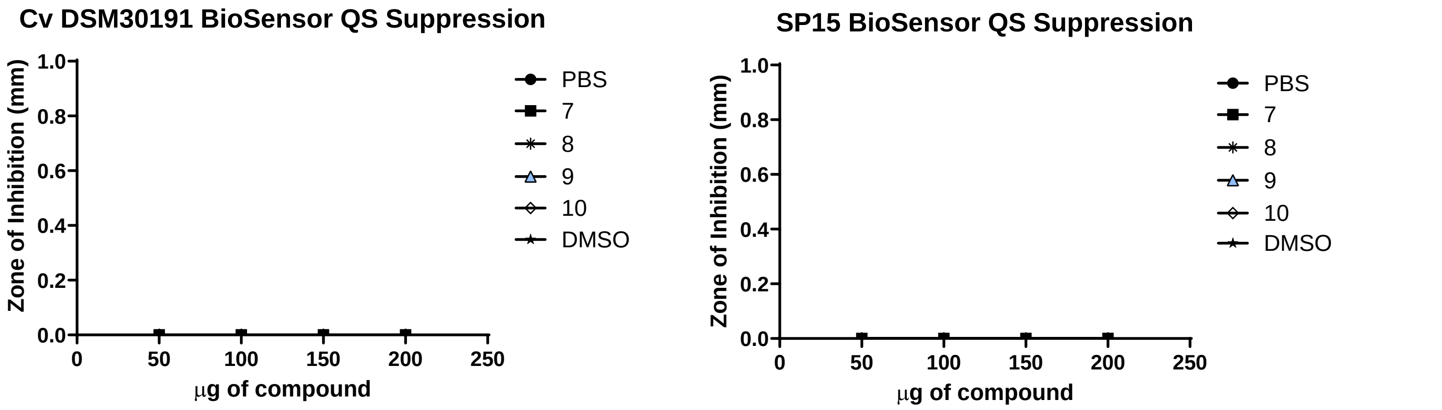
Cv DSM30191 BioSensor QS Suppression
Zone of Inhibition (mm)
1.0
0.8
0.6
0.4
0.2
0.0
0
50
100
150
200
250
μg of compound
PBS
7
8
9
10
DMSO
SP15 BioSensor QS Suppression
Zone of Inhibition (mm)
1.0
0.8
0.6
0.4
0.2
0.0
0
50
100
150
200
250
μg of compound
PBS
7
8
9
10
DMSO

(B)

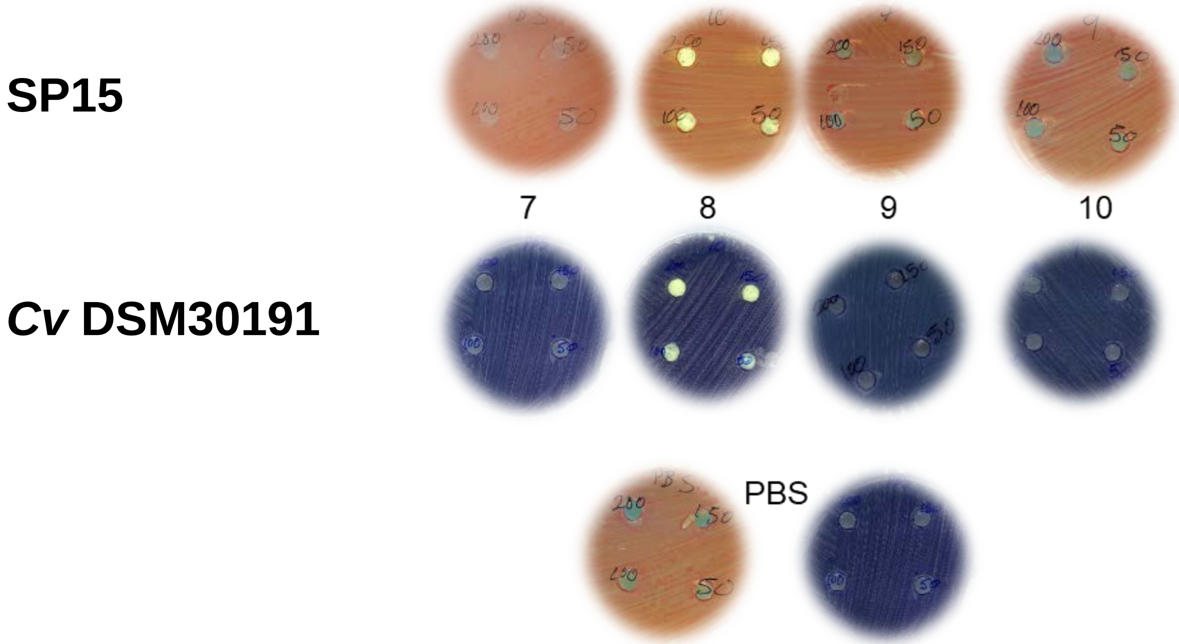
SP15
7
8
9
10
Cv DSM30191
PBS

Figure S9

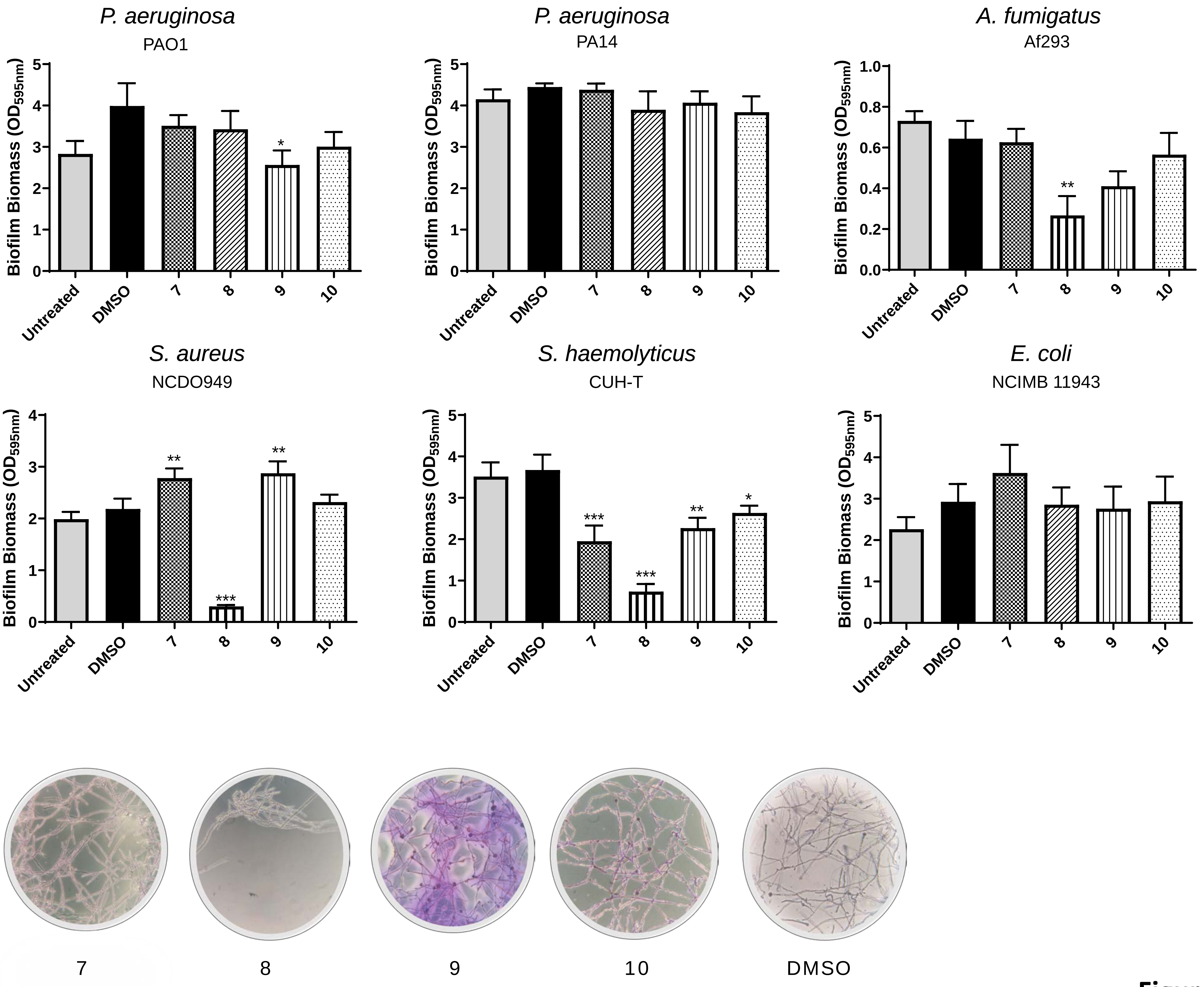

P. aeruginosa
PAO1
P. aeruginosa
PA14
A. fumigatus
Af293
S. aureus
NCDO949
S. haemolyticus
CUH-T
E. coli
NCIMB 11943
Biofilm Biomass (OD595nm)
Untreated
DMSO
7
8
9
10
*
**
***
7
8
9
10
DMSO

Figure S10

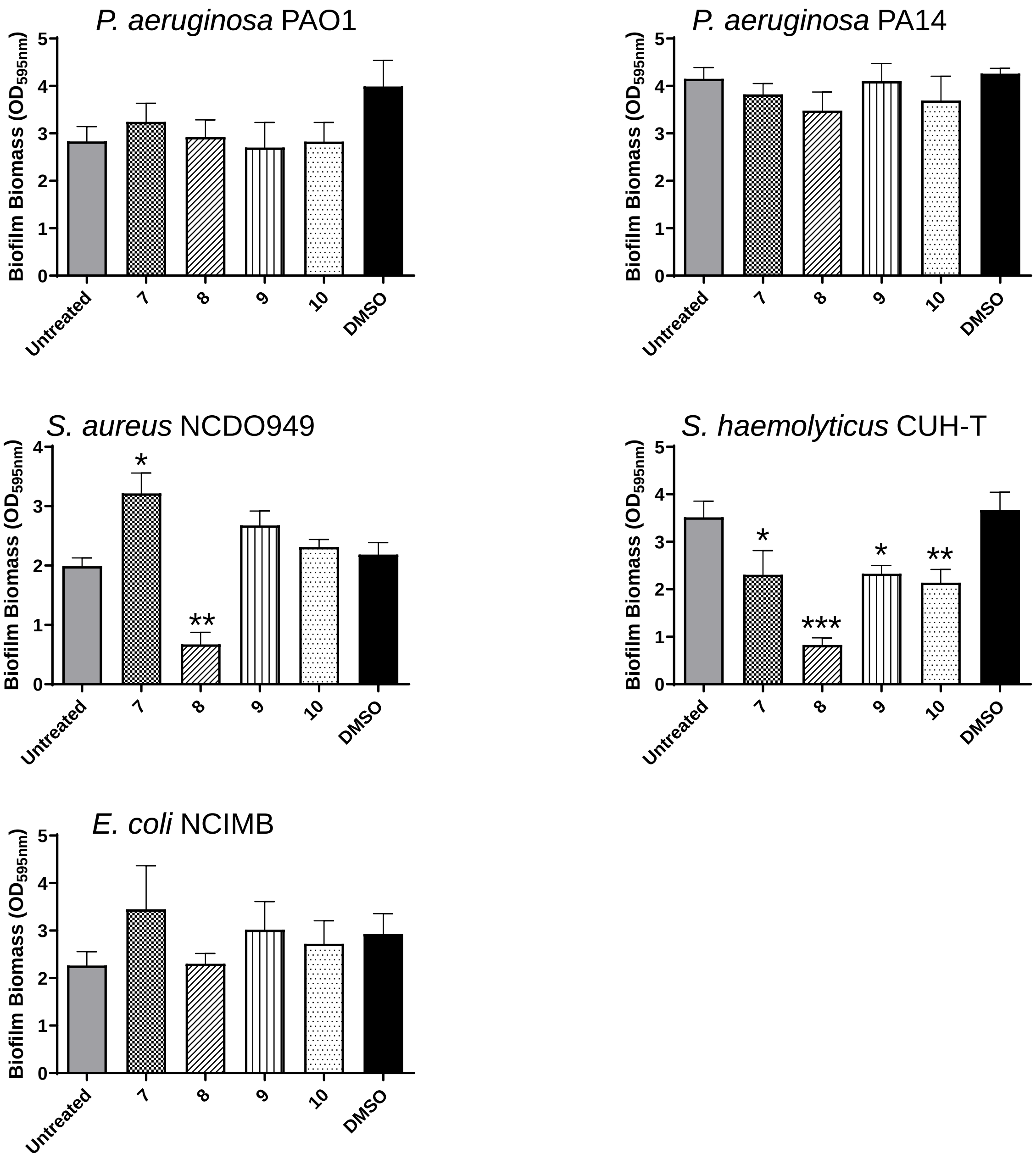
P. aeruginosa PAO1
P. aeruginosa PA14
S. aureus NCDO949
S. haemolyticus CUH-T
E. coli NCIMB
Biofilm Biomass (OD595nm)
0
1
2
3
4
5
Untreated
7
8
9
10
DMSO
*
**
***

Figure S11

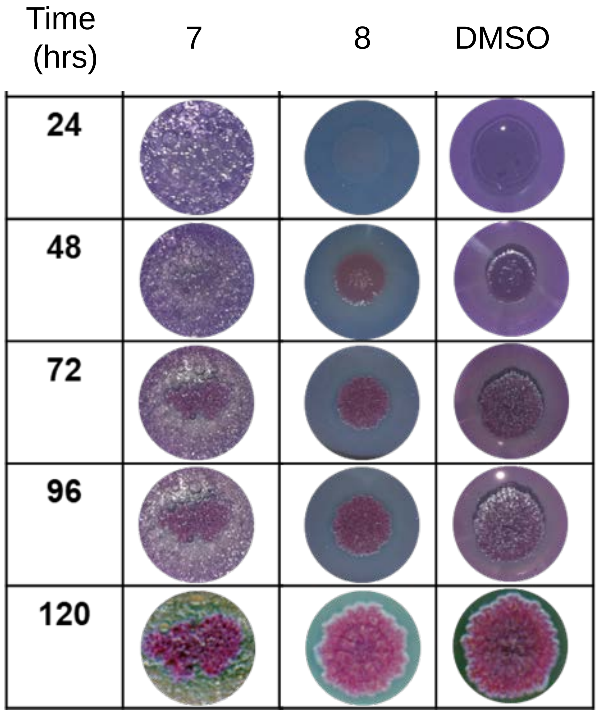

Figure S12

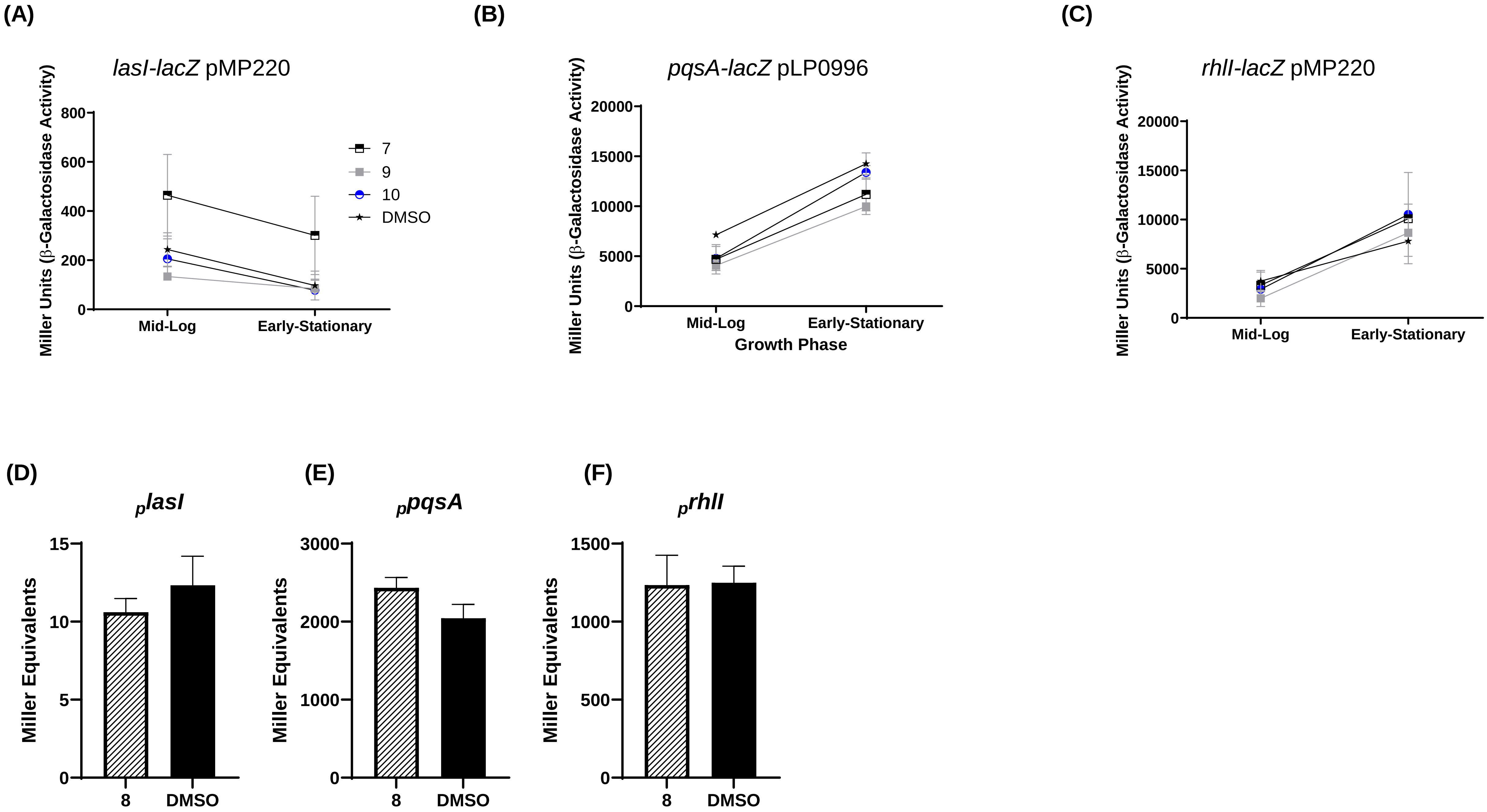
(A)
lasI-lacZ pMP220
Miller Units (β-Galactosidase Activity)
800
600
400
200
0
Mid-Log
Early-Stationary
7
9
10
DMSO
(B)
pqsA-lacZ pLP0996
Miller Units (β-Galactosidase Activity)
20000
15000
10000
5000
0
Mid-Log
Early-Stationary
Growth Phase
(C)
rhlI-lacZ pMP220
Miller Units (β-Galactosidase Activity)
20000
15000
10000
5000
0
Mid-Log
Early-Stationary
(D)
plasI
Miller Equivalents
15
10
5
0
8
DMSO
(E)
ppqsA
Miller Equivalents
3000
2000
1000
0
8
DMSO
(F)
prhlI
Miller Equivalents
1500
1000
500
0
8
DMSO

Figure S13

Supplementary Methods File S1

Molecular modelling of coumarins at the PqsR and LasR receptors

Molecular Docking

The Cartesian coordinates of the two transcriptional regulators from *P. aeruginosa* PqsR (UNIPROT id. Q9I4X0) and LasR (UNIPROT id. P25084) were obtained from the crystallographic structures of PqsR in complex with triazolo-pyridine inverse agonist A (PDB id. 6YIZ) (Schütz *et al.*, 2021) and LasR in complex with 3-oxo-C12-HSL (PDB id. 2UV0) (Bottomley *et al.*, 2007), respectively. In order to perform the docking studies, the co-crystallised molecules were first removed from the structure. Then, the receptors (both proteins) were treated following the standard procedure in AutoDock and the centre of the grid (60 x 60 x 60 Å) (Klett *et al.*, 2012) for the docking was placed where the ligands triazolo-pyridine inverse agonist A (PqsR) and 3-oxo-C12-HSL (LasR) were found experimentally. After computing the grid, the three molecules were docked into each of the proteins using the genetic algorithm (GA) as a searching method implemented in AutoDock4 (Morris *et al.*, 2009). After visual inspection of the docking results, the best solutions were taken. These structures were used for further MD simulation studies.

Molecular Dynamics (MD) simulations

The protonation state of the trititable residues in both PqsR and LasR structures used for the docking studies, were calculated at pH value of 7.0 using the H++ web server. No disulfide bonds were detected in the structures. The protein residues were described with the AMBER force field parameters ff19SB (Tian *et al.*, 2020). The atoms of the small molecules (coumarin, umbelliferone, and esculetin) were described as GAFF/AMBER atom types and the point charges of these molecules were described using a RESP model (using RHF/6-31G∗∗) after geometry optimization (Gaussian16 v.C.01, B3LYP/6-31+G*) (Vosko, Wilk and

Nusair, 1980; Lee, Yang and Parr, 1988; Becke, 1993; Devlin *et al.*, 1995), calculation of the electrostatic potential and point charges derivatisation with antechamber. All proteins were embedded in a truncated octahedron of water molecules (TIP3P) (Jorgensen *et al.*, 1983) and the solvated systems were neutralised using Na+ ions by random substitution of water molecules. For each of the proteins, two different setups were built: (a) the docking solution as a protein monomer with a small single molecule at the binding site; (b) the protein with 10 (PqsR, protein monomer) or 20 (LasR, as protein dimer) small molecules in the solution but not in the binding site from the beginning. Before production, all systems were energetically minimised in three steps, where all protons, the solvent molecules, and finally, the entire system, were gradually relaxed. Then, the system was heated up using the Langevin thermostat (1 ps^{-1} friction coefficient) from 100 to 300 K in 50 ns with a linear increase of the temperature in a NVT ensemble. For this step, all atoms of the solute were restrained by means of application of a harmonic force (constant: 40 kcal mol^{-1} A^{-2}). Finally, the restrains were gradually removed in six steps, where the last two were run under an NPT ensemble. All systems were run in the production phase with a time step of 2 fs. Long-range interactions were calculated using Particle Mesh Ewald summations (Essmann *et al.*, 1995) using Periodic Boundary Conditions and a cutoff of 10 Å for non-bonded interactions. SHAKE algorithm was applied to the water molecules. Those systems derived from the docking (system a) were run for 200 ns. The systems with several molecules in solutions (system b) were carried out for 300 ns. All MD simulations were run using the suite of programs Amber20 (Case *et al.*, 2023) and the MD trajectories were analysed using cpptraj v5.1.0. (Roe and Cheatham, 2013).

Pocket and tunnel screening using CAVER

Caver web server was used to determine, first possible pockets in the apo form of both proteins (Stourac *et al.*, 2019). For our study, we use an equilibrated structure of each of the

receptors in water solution. From the several pockets detected, we selected the one where the co-crystallised ligands were found. Starting from these pockets, Caver determines possible tunnels to access the cavity. Caver default parameters were used in all cases.

Supplementary Methods File S2

Chemistry Experimental

General information

Solvents and reagents were used as obtained from commercial sources and without purification, unless stated otherwise.

Wet flash column chromatography was carried out using Kieselgel silica gel 60, 0.040-0.063 μm (Merck). TLC was carried out on pre-coated silica gel plates (Merck 60 PF254). Visualisation was achieved by UV light, unless otherwise stated.

Melting points were obtained on a uni-melt Thomas Hoover Capillary melting point apparatus.

IR spectra were recorded on Perkin-Elmer FT-IR Paragon 1000 spectrophotometer. Solid samples dissolved in dichloromethane (DCM), dispersed as thin films on NaCl plates and the DCM was allowed to evaporate before measurement of sample.

NMR spectra were run in $CDCl_3$ using TMS as the internal standard at 25 °C unless otherwise specified. ^1H NMR (400 MHz) spectra and ^1H NMR (300 MHz) spectra were recorded on Bruker Avance 400, and Bruker Avance 300 NMR spectrometers respectively. ^{13}C (100 MHz) spectra, and ^{13}C (75.5 MHz) spectra were recorded on Bruker Avance 400, and Bruker Avance 300 NMR spectrometers respectively in proton decoupled mode. ^{19}F (376 MHz) spectra, and ^{19}F NMR (282 MHz) spectra were recorded on a Bruker Avance 400 NMR spectrometer and Bruker Avance 300 NMR spectrometer in proton decoupled mode. All spectra were run at University College Cork. Chemical shifts (δ) are expressed as parts per million (ppm), positive shift being downfield from TMS; coupling constants (*J*) are expressed in hertz (Hz). Splitting patterns in ^1H NMR spectra are designated as s (singlet), bs (broad singlet), d (doublet), dd (doublet of doublets), dt (doublet of triplets), t (triplet), q (quartet), quin (quintet), sext (sextet), sept (septet), and m (multiplet). For ^{13}C NMR spectra, the number of attached protons for each signal was determined using the DEPT pulse sequence run in the DEPT-90 and DEPT-135 modes; carbons without attached protons have been designated qC. COSY, HSQC and HMBC experiments were performed to aid the NMR assignment. Compounds were numbered for ease of assignment and do not necessarily represent IUPAC nomenclature.

The Microanalysis Laboratory, National University of Ireland, Cork, performed elemental analysis using a Perkin-Elmer 240 and Exeter Analytical CE440 elemental analysers.

Synthesis of Aryl Fluorosulfates

2-Oxo-2H-chromen-6-yl fluorosulfate (1)

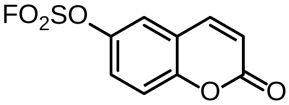

Compound **1** was prepared using a 20 mL, two-chamber, COware reactor. Chamber A was filled with 1,1'- sulfonyldiimidazole (297 mg, 1.5 mmol, 1.5 equiv.) and potassium fluoride (232 mg, 4.0 mmol, 4.0 equiv.). Chamber B was then filled with 6-hydroxycoumarin (162 mg, 1 mmol, 1.0 equiv.), triethylamine (279 µL, 2.0 mmol, 2.0 equiv.) and DCM (4 mL). The chambers were then sealed before 1 mL trifluoroacetic acid was added by injection through the septum in chamber A and instant gas formation was observed. After 18 hours stirring at 25˚C, one of the caps was removed to release the residual pressure. The reaction was stirred for another 15 minutes to ensure that all sulfuryl fluoride was extracted out of the fume hood. Next, the contents of chamber B, as well as DCM washings of chamber B were passed through Celite. The DCM was removed under reduced pressure .The crude compound was purified by silica gel column chromatography (hexane/EtOAc, (1:1)) to give pure compound **1** as a yellow solid (238 mg, 97%); m.p. 86-90 °C (lit. 85-88°C); ν_{max}/cm^{-1} (NaCl): 1774 (C=O stretch), 1637 (aromatic C=C bend), 1383 (S=O stretch). ^{1}H NMR (400 MHz, $CDCl_3$) δ: 7.71 (1H, d, *J* = 9.6 Hz, C**H**), 7.55 – 7.48 (2H, m, C**H** & C**H**)) 7.47 – 7.42 (1H, m, C**H**) 6.56 (1H, d, *J* = 9.7 Hz, C**H**) ppm; ^{13}C NMR (100 MHz, $CDCl_3$) δ: 159.4 (q**C**), 153.2 (q**C**), 145.6 (q**C**OSO_2F), 141.9 (**C**H), 124.3 (bs, **C**H), 120.0 (bs, **C**H), 119.9 (q**C**), 119.1 (**C**H), 118.8 (**C**H) ppm; ^{19}F NMR (376 MHz, $CDCl_3$) δ: 37.7 ppm; Anal. calculated for $C_9H_5O_5FS$: C, 44.27, H, 2.06. Found: C, 44.64, H, 2.15.

Spectral characteristics are consistent with previously reported data (Guo *et al.*, 2018).

^{1}H NMR, ^{19}F NMR and ^{13}C NMR Spectra

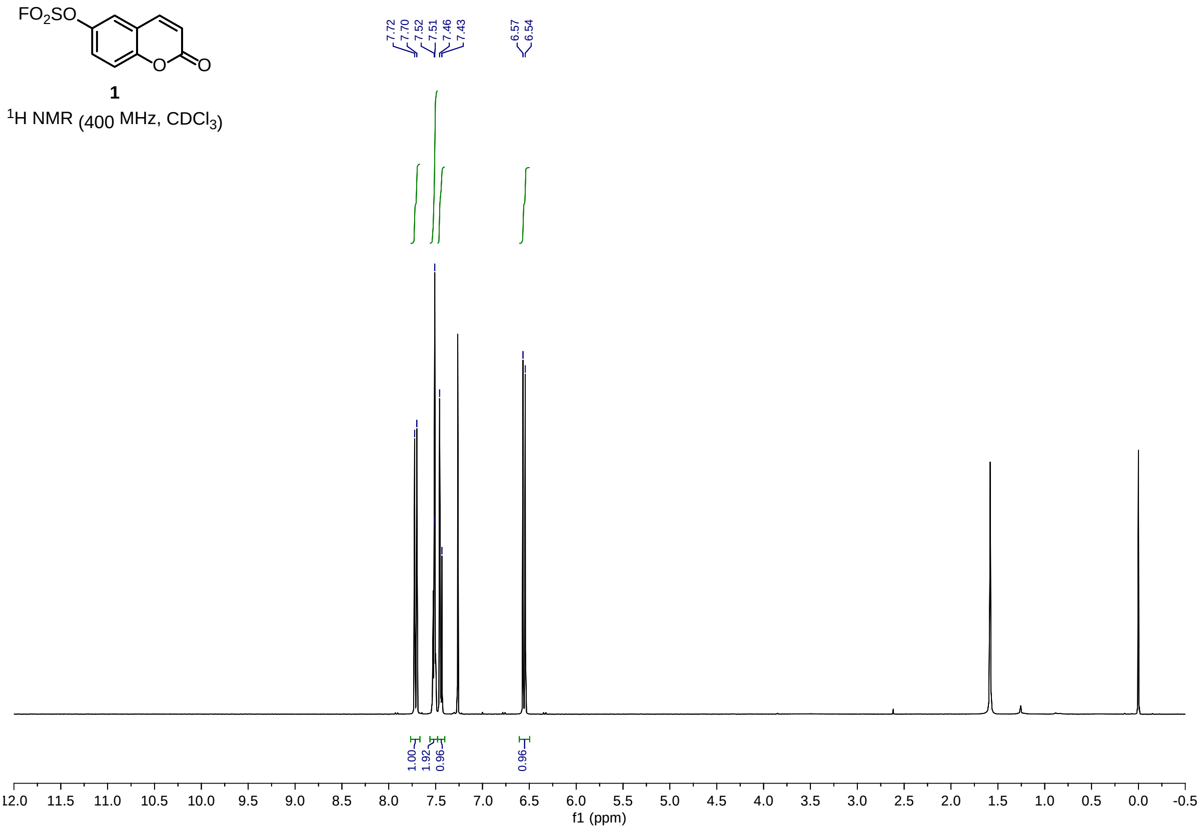

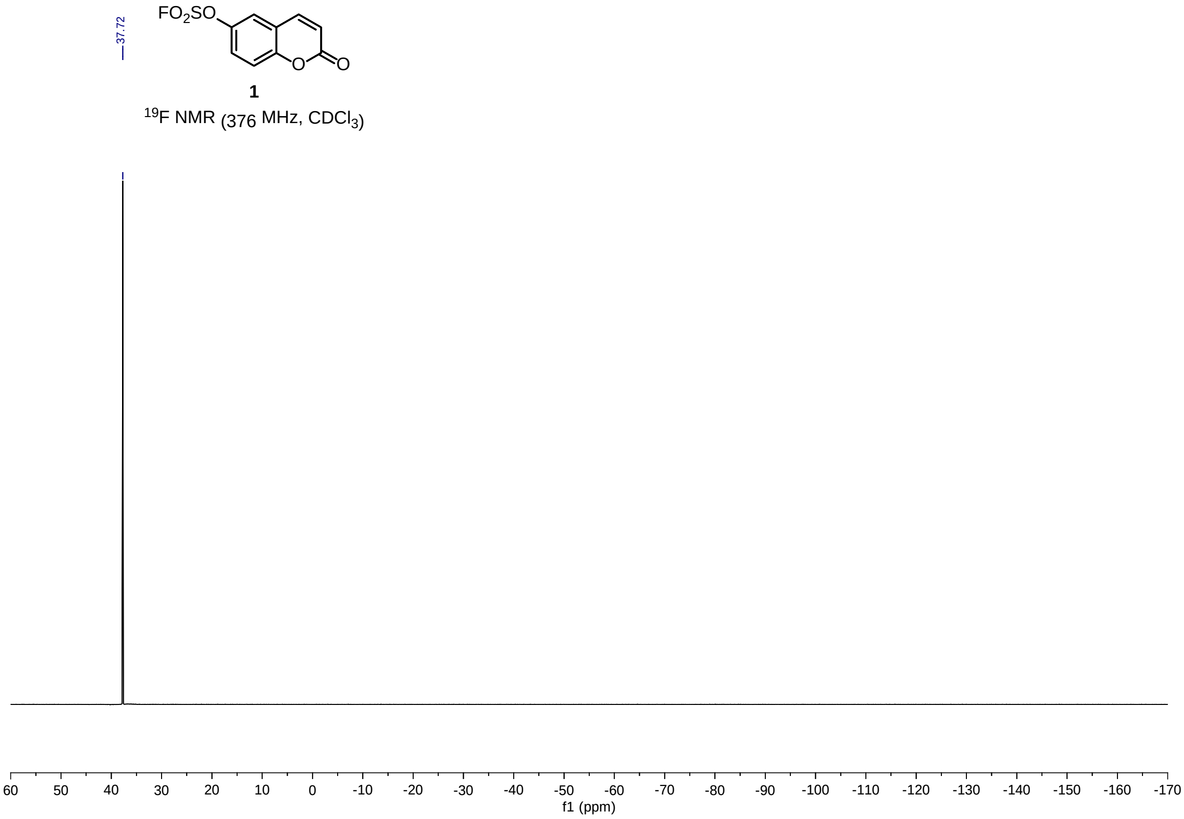

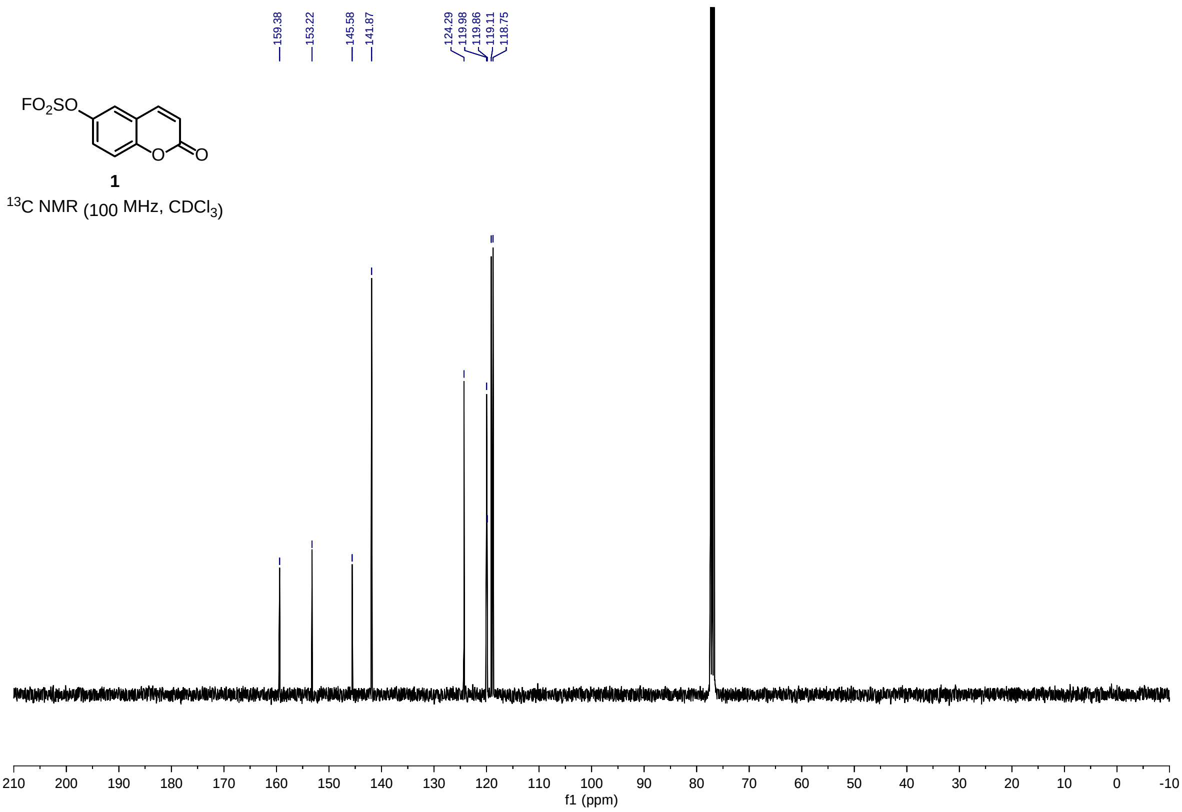
159.38
153.22
145.58
141.87
124.29
119.98
119.86
119.11
118.75
FO2SO
O
O
1
13C NMR (100 MHz, CDCl3)
210
200
190
180
170
160
150
140
130
120
110
100
90
80
70
60
50
40
30
20
10
0
-10
f1 (ppm)

Supplementary File References

Baysse, C., Cullinane, M., Denervaud, V., Burrowes, E., Dow, J.M., *et al*. (2005) Modulation of quorum sensing in *Pseudomonas aeruginosa* through alteration of membrane properties. Microbiology **151**: 2529–2542.

Guo, T., Meng, G., Zhan, X., Yang, Q., Ma, T., Xu, L., Sharpless, K.B., Dong, J. (2018). A New Portal to SuFEx Click Chemistry: A Stable Fluorosulfuryl Imidazolium Salt Emerging as an “F−SO 2 +” Donor of Unprecedented Reactivity, Selectivity, and Scope. *Angew Chem Int Ed* **57(10)**: 2605–2610.

Holloway, B.W. (1955). Genetic recombination in *Pseudomonas aeruginosa*. *J Gen Microbiol* **13**: 572-581.

Liberati, N.T., Urbach, J.M., Miyata, S., Lee, D.G., Drenkard, E., Wu, G., Villanueva, J., Wei. T., Ausubel. F.M. (2006). An ordered, nonredundant library of *Pseudomonas aeruginosa* strain PA14 transposon insertion mutants. *Proc Natl Acad Sci USA* **103(8)**:2833-8.

Mathee, K. (2018). Forensic investigation into the origin of *Pseudomonas aeruginosa* PA14—old but not lost. *J Med Microbiol* **67**: 1019–1021.

McGrath, S., Wade, D.S., Pesci, E.C. (2004). Dueling quorum sensing systems in *Pseudomonas aeruginosa* control the production of the Pseudomonas quinolone signal (PQS). *FEMS Microbiol Lett* **230(1)**: 27-34.
